# Bone marrow mesenchymal stem cells combined with estrogen synergistically promote endometrial regeneration and reverse EMT via Wnt/β-catenin signaling pathway

**DOI:** 10.1186/s12958-022-00988-1

**Published:** 2022-08-15

**Authors:** Liwei Yuan, Jia Cao, Mingyue Hu, Dabao Xu, Yan Li, Shiyun Zhao, Juanjuan Yuan, Huixing Zhang, Yani Huang, He Jin, Meixia Chen, Dan Liu

**Affiliations:** 1 Department of Gynecology, General Hospital of Ningxia Medical University, Yinchuan, Ningxia China; 2 College of Clinical Medicine, Ningxia Medical University, Yinchuan, Ningxia China; 3 Department of Beijing National Biochip Research Center Sub-Center in Ningxia, General Hospital of Ningxia Medical University, Yinchuan, Ningxia China; 4 Department of Gynecology, Third Xiangya Hospital of Central South University, Changsha, Hunan China; 5 Key Laboratory of Ministry of Education for Fertility Preservation and Maintenance, Ningxia Medical University, Yinchuan, Ningxia China

**Keywords:** Intrauterine adhesion, Bone marrow mesenchymal stem cells, Estrogen, Epithelial-mesenchymal transition, Wnt/β-catenin pathway

## Abstract

**Background:**

Intrauterine adhesion (IUA) is a clinical disease characterized by the uterine cavity occlusion caused by the damage of the endometrial basal layer. Bone marrow mesenchymal stem cells (BMSCs) transplantation have the potential to promote endometrial regeneration mainly through paracrine ability. Estrogen is an indispensable and important factor in the repair of endometrial damage, which has been reported as a promising and adjunctive therapeutic application for stem cell transplantation therapy. This study aims to investigate the synergistic effect of BMSCs and estrogen on improving the endometrial regeneration and restoring the endometrium morphology in a dual damage model of IUA in rabbits and the underlying molecular mechanisms.

**Methods:**

BMSCs were isolated and identified by adipogenic and osteogenic differentiation and flow cytometry assays. The rabbit IUA animal model was established by a dual damage method of mechanical curettage and lipopolysaccharide infection. Additionally, we investigated the therapeutic impact of both BMSCs and estrogen either separately or in combination in a rabbit model. The retention of PKH26-labeled BMSCs was observed by vivo fluorescence imaging.The number of endometrial glands and the degree of fibrosis were observed by H&E and Masson staining respectively. Western blotting, Immunohistochemistry and immunofluorescence staining were performed to detect biomarkers related to endometrial epithelium, endometrial fibrosis and EMT. Finally, the protein expression of core molecules of Wnt/β-catenin pathway was detected by Western blotting.

**Results:**

PKH26-labeled fluorescence results revealed that BMSCs appeared and located in the endometrial glands and extracellular matrix area when orthotopic transplanted into the uterine cavity. Histological assays showed that remarkably increasing the number of endometrial glands and decreasing the area of endometrial fibrosis in the BMSCs combined with estrogen treatment group. Moreover, downregulated expression of fibrosis markers (fibronectin, CollagenI, a-SMA) and interstitial markers (ZEB1, Vimentin, N-cadherin), as well as upregulated E-cadherin expression were found in the combined group. Further study of in vivo staining revealed that fluorescence intensity of CK7 was stronger in the combined group than that of direct BMSCs intrauterine transplantation, while vimentin showed the opposite results. Moreover, the protein levels of β-catenin, Axin2, C-myc, CycinE of Wnt/β-catenin signaling pathway increased in the BMSCs combined with estrogen group than in the other treatment groups.

**Conclusion:**

BMSCs combined with estrogen can promote the differentiation of stem cells into endometrial epithelial cells to facilitate the regeneration of damaged endometrium. The potential mechanism of the synergistic effect may inhibit the occurrence of EMT by activating the Wnt/β-catenin signaling pathway.

**Supplementary Information:**

The online version contains supplementary material available at 10.1186/s12958-022-00988-1.

## Introduction

Intrauterine adhesion (IUA) is caused by trauma and infection to damage the endometrial basal layer. The main clinical manifestation of IUA is partial or complete loss of functional endometrium, resulting in endometrial fibrosis or scar formation [1, 2], causing a series of clinical symptoms such as abnormal menstruation, infertility, miscarriage and pregnancy abnormalities [3, 4]. At present, hysteroscopic adhesiolysis combined with postoperative hormone therapy are mainly used to stimulate endometrial regeneration from residual progenitor cells and endometrial stem cells [5]. However, prognosis of moderate-to-severe IUA still poor because of the high recurrence and readhesion [6]. Thus, it is necessary to explore more effective therapeutic methods to repair the injured endometrium and improve the endometrial function.

Recent advances highlight therapeutic strategies with stem cells transplantation as an alternative choice for the IUA treatment [7]. Bone marrow mesenchymal stem cells (BMSCs) is widely used in the treatment of endometrial diseases because of its easy separation, low immunogenicity and multiple differentiation potential [8, 9]. Currently, a variety of biomaterials have been used to assist BMSCs migrate to the damaged endometrial area to effectively promote endometrial regeneration [10, 11]. Zheng et al. [12] provided specific evidences suggesting that CM-Dil-labeled MSCs can be distributed in the endometrial stroma and epithelial area after transplantation. Xia et al. [13] reported that electroacupuncture plays an important role in supporting BMSCs in the repair of thin endometrium by promoting the paracrine effect of BMSCs. Xiao et al.found that PGS scaffolds promote differentiation of BMSCs restoring the morphology and function of wounded rat uterus [14].

Estrogen has been commonly used as an important adjuvant therapy to prevent postoperative re-adhesion. A recent study has shown that estrogen combined with hAMSC transplantation can effectively promote hAMSC differentiation to generate endometrial epithelial cells through Notch signaling [1]. Previous studies have reported that a combination of BMSCs with estrogen could promote endometrial repair [15, 16]. However, it was only a preliminary study on the therapeutic effect of combined application for IUA, the specific underlying mechanism remain unclear. It is highlighted that epithelial-mesenchymal transition (EMT), one of the most important mechanisms of fibrotic diseases, has been considered to be intimately involved in the pathogenesis of IUA [17]. Based on a report indicating that mesenchymal protein vimentin increased while epithelial marker cytokeratin decreased in IUA mouse models [18]. Yao et al. [11] also reported that BMSCs derived exosomes promoted endometrium recovery by reversing EMT via targeting the TGF-β1/Smad pathway. It is suggested that inhibiting EMT may be therefore a novel strategy for treatment of IUA. Meanwhile, increasing evidences have shown that the canonical Wnt/β-catenin signaling pathway is closely related to the occurrence of fibrosis [19] as well as involved in regulating the biological functions of BMSCs [20]. It was proved that sex hormones regulate Wnt pathway to maintain endometrial homeostasis [21]. The above findings clearly illustrated that targeting Wnt/β-catenin pathway will be a promising strategy to prevent the development of IUA.

In the present study, we aimed to explore the molecular mechanism of estrogen combined with BMSCs transplantation in promoting the regeneration of injured endometrium in IUA. The results showed that BMSCs cooperated with estrogen may promote the differentiation of stem cells into endometrial epithelial cells and inhibit the occurrence of EMT via Wnt/β-catenin pathway. Our findings provide a new perspective for the treatment of IUA.

## Materials and methods

### Experimental animals

All the animals were purchased from the experimental animal center of Ningxia Medical University. The 3-week-old female New Zealand white rabbits were used for the isolation of BMSCs and the rabbits aged 12 weeks were used for the establishment of the IUA model. The experimental protocols and animal handling procedures were approved by the Ethics Committee of General Hospital of Ningxia Medical University.

### Isolation, identification and differentiation of BMSCs

BMSCs were isolated from the tibia and femur of 3-week-old female New Zealand white rabbits using the method of adherent culture of whole bone marrow. Briefly, under complete aseptic conditions, bone marrow cells were harvested and cultured with low glucose Dulbecco’s Modified Eagle’s Medium (DMEM) (Gibco, Gainesville, MD, USA) containing 10% fetal bovine serum (FBS) (Gibco, Australia) and 1% penicillin–streptomycin (Gibco, USA) at 37℃ in a humidified atmosphere with 5% CO2. The medium was refreshed every 2 days until the cell confluence reached 90%. Then, the cells were digested with 0.25% trypsin (Hyclone, USA) and subcultured to the third generation for following experiment. Subsequently, BMSCs were washed twice with PBS and fixed in 4% para formaldehyde (PFA), and then the related cell-surface markers such as CD29, CD44, CD34 and CD45 (Bioss, USA) were evaluated by flow cytometer.

Next, the osteogenesis and adipogenesis differentiation experiments of BMSC were performed. Briefly, When the degree of cell confluence reached 80%, the cells were digested with 0.25% trypsin and seeded into a 0.1% gelatin-coated culture dish, then add 2 mL of commercial osteogenic or adipogenic differentiation medium of induction kits according to the manufacturer’s instructions (RBXMX-90021 and RBXMX-90031, Cyagen, Santa Clara, CA). The medium was changed every 3 days and observe the cell morphology and growth for 3 weeks. The mineral components are stained with Alizarin Red and the lipid accumulation are stained with Oil Red O. Observe the effect of osteogenic or adipogenic induction under the microscope.

### PKH26 fluorescent dye-labeled BMSCs

To track the distribution of BMSCs after transplantation, cells were harvested and labeled with PKH26, a lipophilic red fluorescent linker dye (Cat. # MINI26, Sigma Aldrich, USA). Add culture medium containing 1 ml Diluent C and 4ul PKH26 reagent when BMSCs reach 80% confluency after centrifuged and washed. Then, the mixture was mixed in a centrifuge tube and incubated at room temperature for 5 min. Subsequently, 2 mL of FBS was added to stop the staining. The supernatant was discarded and cells were resuspended in fresh medium. The PKH26-labeled BMSCs were examined under fluorescent microscope (Olympus, Tokyo, Japan). The cells were maintained in the growth medium prior to being transplantated into the uterine cavity of rabbits.

### Animal IUA model and experimental protocol

The IUA rabbit model was generated by a dual damage method of mechanical curettage and lipopolysaccharide (LPS, 6 mg/L, Sigma) infection. Briefly, After anesthesia with 3% sodium pentobarbital (1 mL/kg), the abdominal wall and cavity was surgically opened with a vertical incision to expose the bilateral uterus under sterile conditions. A 2 mm transverse incision at the bilateral uterus junctions was cut with ophthalmic scissors, and a micro endometrial curette was inserted into the uterine cavity through the incision and rotated repeatedly until the uterine wall appeared rough and grainy. Subsequently, the end of the LPS-soaked cotton thread was placed into the uterine cavity through the uterine incision, leaving about 5 cm of the other end outside, and remove the cotton thread after 48 h. Finally, the abdominal cavity were stitched in layers and injected penicillin to prevent infection.

The study comprised of 75 female New Zealand white rabbits aged 12 weeks, which were randomly assigned to the following five groups (15 in each group): sham operation group (sham), rabbits merely had laparotomy without any treatment. IUA model group (IUA), rabbits underwent induction of previously described double injury. estrogen treated group (IUA + E2), intramuscular injections of estrogen (0.1 mg/kg) for 21 days. BMSCs treated group (IUA + BMSCs), 7 × 106 PKH26-labeled BMSCs were resuspended in 200μL medium and orthotopic injected into the bilateral injured uterine segment. BMSCs plus estrogen group (IUA + BMSCs + E2). All the study rabbits were euthanized on the 1 week, 2 weeks, 3 weeks and bilateral uterus were collected for the next experiment.

### Hematoxylin–eosin (H&E) staining and Masson staining

At the appointed time, rabbits were sacrificed and bilateral uterine were resected. The endometrial tissue samples were fixed in 4% paraformaldehyde (Sigma-Aldrich, Cat# P6148) for 24 h and then embedded into paraffin blocks after dehydration and hyalinization. The paraffin-embedded sections were cut into 4-μm serial sections and subjected to HE and Masson staining according to routine procedures. Sections were taken using an orthostatic microscope (Olympus, Tokyo, Japan). Choose three different high-magnification fields, and calculate the number of endometrial glands and the degree of fibrosis by HE and Masson staining respectively. Image J (Image in Java, USA) software was used for statistical analysis of the average proportion of each group.

### Immunohistochemistry staining

Samples were fixed in 4% paraformaldehyde and embedded in paraffin. The transverse paraffin sections were deparaffinized using xylene, rehydrated through a series of alcohol gradients. Then, the sections were incubated in 3% hydrogen peroxide for 30 min to inactivate endogenous peroxidase activity and incubated with the following primary antibodies: anti-p-Keratin (1:200, Abcam, USA,) and anti-a-SMA (1:200, Abcam, USA) for 2 h. Subsequently, the sections were washed with PBS treated with anti-rabbit or anti-mouse immunoglobulinG (IgG) secondary antibodies. Protein expression was visualized with diaminobenzidine (Dako Cytomation, USA) staining. The number of positively stained cells were observed under a high-power microscope and quantified at five randomly selected fields.

### Immunofluorescence staining

To further verify the effects of BMSC combined with estrogen therapy on the growth of endometrial epithelial cells and endometrial stromal cells, we detected the expression of epithelial specific marker CK7 and interstitial specific marker Vimentin by Immunofluorescence staining. Uterine tissues were fixed with 4% paraformaldehyde for 24 h and then was dehydrated in 30% sucrose solution at 4 °C. The dehydrated tissues were embedded with OCT compound (Tissue-Tek; Sakura) for rapid freezing, and cut into 6-μm slices with a microtome. Sections were incubated with blocking solution consisting of 0.3% Triton-X 100 and 5% donkey serum, followed by incubation with antibodies against CK7 (1:200, Abcam, USA) and Vimentin (1:200, Abcam, USA) at 4 °C overnight. After the slices was washed with PBS solution and incubated with Alexa fluorochrome-conjugated antibodies (1:200, Life Technologies, USA) at room temperature for 2 h. The nucleus was visualized with DAPI staining (1:500, Sigma, USA) for 15 min in the dark. Images were taken under a fluorescence microscope (Olympus, Japan).

### Western blot analysis

The total protein samples was extracted and lysed from rabbit uterine tissues using RIPA lysis buffer (Solarbio, China), supplemented with the protease inhibitors (1 μg/mL) and PMSF (100 mM) and then homogenized using sterile scissor. The extract was centrifuged at 12,000 rpm for 20 min after lysising on ice for 1 h, and then the supernatant was collected and the total protein concentration was determined using the BCA protein assay kit (Thermo Scientific, MA, USA). Protein samples (30 μg) was separated on a 10% SDS-PAGE gel and transferred to PVDF membranes (Millipore, MA, USA). The membranes were blocked with 5% defatted milk for 1 h at room temperature and incubated overnight with the following primary antibodies at 4 °C: anti-N-cadherin (1:1000, Abcam), anti-Collagen I (1:1000, Abcam), anti-TGF-β1 (1:1000, CST), anti-a-SMA (1:2000, Abcam), anti-E-cadherin (1:1000, CST), anti-Vimentin (1:2000, Abcam), anti-ZEB1 (1:1000, Santa Cruz), anti-β-catenin (1:1000, NOVUS), anti-C-myc (1:1000, Abcam), anti-CyclinE (1:1000, Abcam), anti-Axin2 (1:2000, Abcam), anti-GAPDH (1:5000, Abcam). Membranes were washed three or four times with PBS and then incubated with the secondary antibody at 37℃ for 1 h in the dark. Finally, the proteins were visualized using ECL in the BioImaging System (BIO-RAD, USA). GAPDH was used as an internal control to normalize the relative expression of each protein of interest. The density of the bands was analyzed using ImageJ software.

### Statistical analysis

Statistical analysis was performed using SPSS version 20.0 and GraphPad Prism version 6.0. All results were repeated 3 times and presented as the mean ± SD. Two samples were compared using independent sample two-tailed t-test. Statistical differences among multiple groups were determined by one-way analysis of variance (ANOVA). P < 0.05 was considered to be statistically significant.

## Results

### In vitro culture and phenotypic identification of BMSCs

**Fig. 1:**
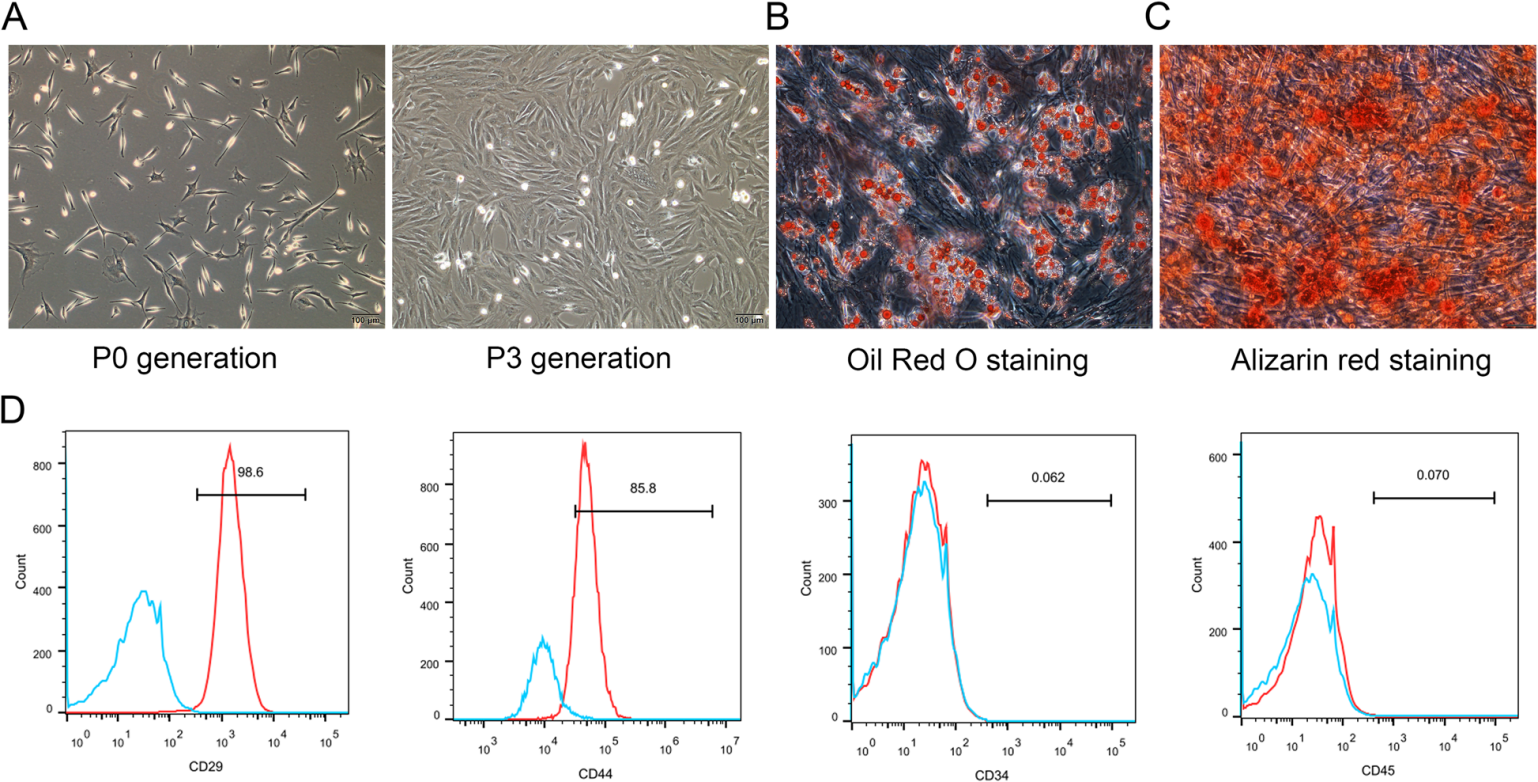
Culture, differentiation and identification of BMSC in vitro. **A** Cell morphology of P0 and P3 generations of BMSCs (× 100, scale bar = 100 μm). **B** Adipogenic differentiation of BMSCs were identified by Oil red O staining in three weeks (× 200, scale bar = 50 μm). **C** Osteogenic differentiation of BMSCs were identified by Alizarin red staining in three weeks (× 200, scale bar = 50 μm). **D** Flow cytometric analysis of BMSCs. BMSCs were positive for CD29 and CD44 and negative for CD34 and CD45

In order to identify the characteristic and phenotypic of BMSCs, primary cell culture and surface marker detection were performed. The morphology of primary cultured cells was a long shuttle shape and evenly distributed adherent growth. When BMSCs were expanded and passaged to the third generation, the cells tended to be stable and exhibited a fibroblast-like morphology, and arranged in a spiral pattern (Fig. 1A). Then, adipogenic and osteogenic differentiation of BMSCs were identified, and the results showed that after cultured with corresponding differentiation media for three weeks, the cell morphology changed from the original long spindle to round, a large number of vacuoles appeared in the cytoplasm, Oil Red O staining found a lipid droplets formed in the cells, indicating that BMSCs could differentiated into adipocytes (Fig. 1B). Also, Alizarin red staining showed a calcified extra-cellular matrix appeared in the cells, indicating BMSCs could differentiate into osteogenic cells (Fig. 1C). Then, cell surface phenotypings were detected by flow cytometry. As shown in Fig. 1D, the cells presented positive expression of the CD29 and CD44, whereas negative expression of CD34 and CD45. These results were consistent with the characteristics of mesenchymal stem cells.

### The migration of PKH26-labeled BMSCs

**Fig. 2:**
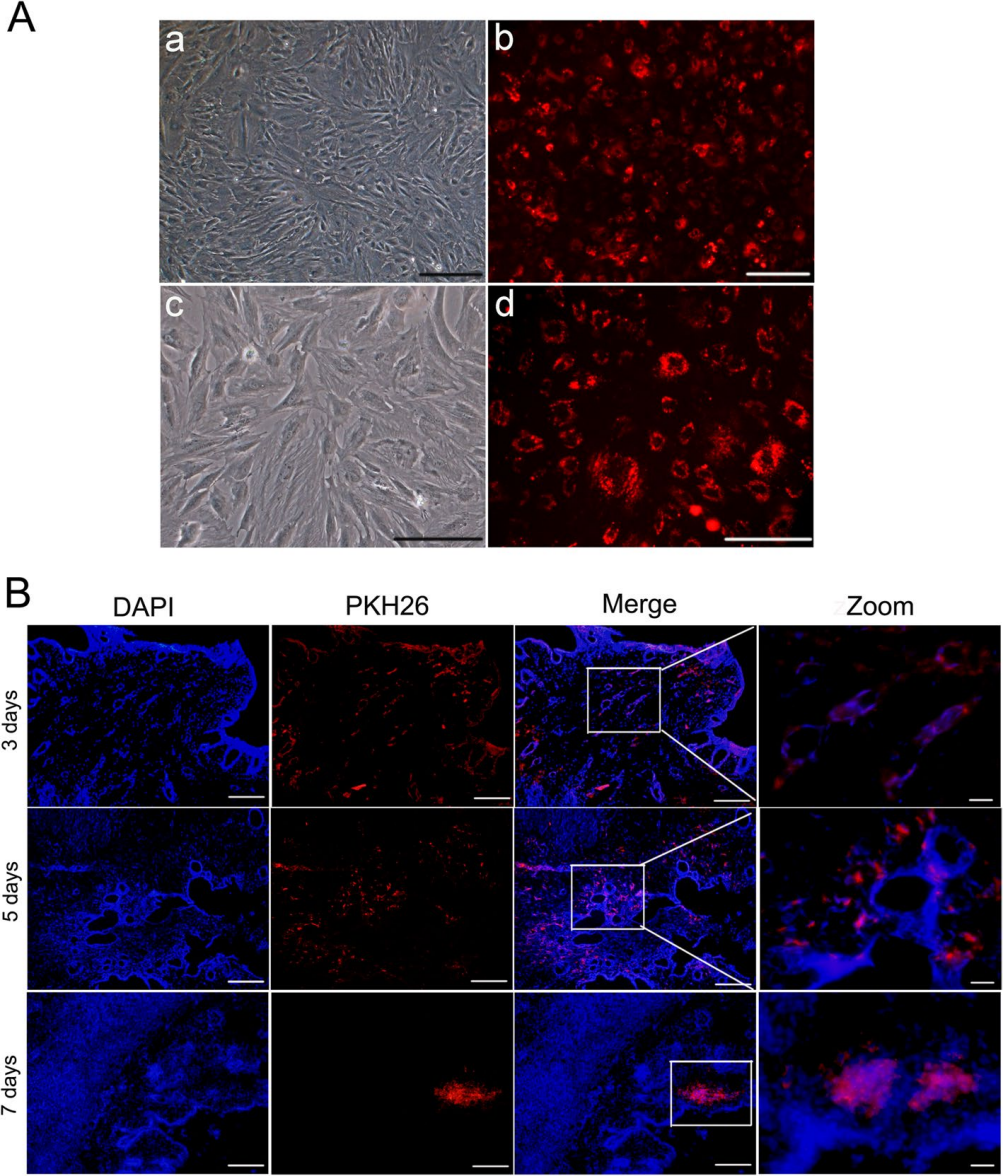
PKH26-labeled BMSCs tracing in vivo. **A** PKH26-labeled BMSCs were observed under a fluorescence microscope (a,b: × 40, scale bar = 200 μm; c,d: × 100, scale bar = 100 μm). **B** Immunofluorescence staining for the distribution of pKH26-BMSCs after orthotopic transplantationy (Merge: × 100, scale bar = 100 μm; Zoom: × 400, scale bar = 20 μm)

To track and determine whether BMSCs could migrate to the damaged endometrium to repair tissue regeneration, P3 generation cells to be labeled with the red fluorescent cell membrane dye PKH26 and transplanted into the IUA model. The positive rate of PKH26-labeled cells reached 80% under a fluorescence microscope (Fig. 2A). In addition, the frozen OCT-embedded uterine tissue sections were observed and found that there were a large number of scattered red fluorescence distributed around the endometrial glands at 3 days after transplantation. And there were still more red fluorescence, mainly located in a small number of glands and surrounding matrix after orthotopic transplantation 5 days. With the extension of transplantation time to 7 days, the red fluorescence gradually decreased and mainly distributed in the endometrial stromal area (Fig. 2B). The fluorescence intensity was very weak in the uterine area at 2 weeks after transplantation. These results indicate that PKH26 can successfully label BMSCs and can be used to track the distribution of BMSCs in vivo.

### Quantitative assessment of BMSCs transplantation time

**Fig. 3:**
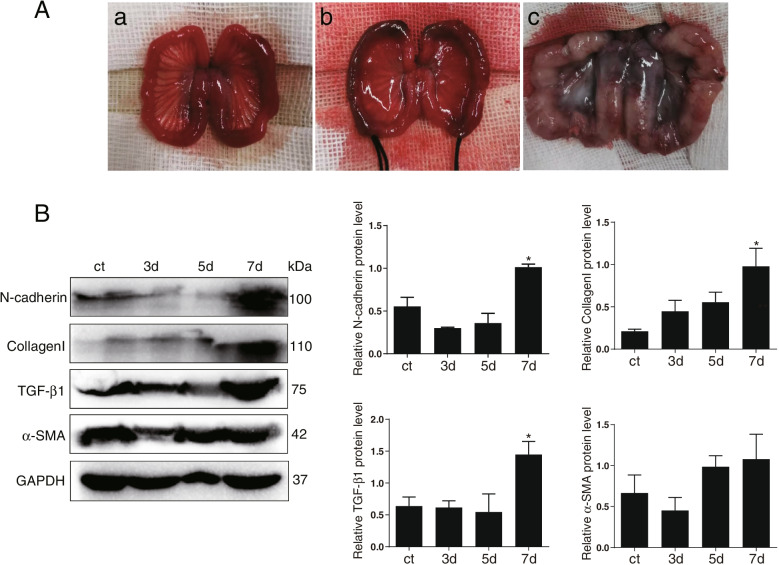
Quantitative assessment of BMSCs transplantation time. **A** (a): The normal uterine morphology of a rabbit; (b): Morphology of rabbit uterus after mechanical and infectious double injury; (c): Morphology after one week of injury). **B** Western blot was used to detect the protein expression of fibrosis markers (N-cadherin, Collagen I, TGF-β1, α-SMA) at different time points. ^*^p < 0.05. The explanation for the cropping of gels and blots in the experiment is as follows: the same proteins were separated by electrophoresis and transferred to PVDF membrane. According to the different molecular weight of the target antibody, the protein on a PVDF membrane was cropped horizontally. Then, the corresponding antibody was incubated and exposed, and the position of antibody was indicated by protein marker to ensure the exclusion of interference from non-specific antibody magazines

In order to explore the best time for the evaluation of the therapeutic effect of subsequent BMSCs transplantation, IUA animal model was constructed by mechanical and infection double injury method (Fig. 3A). Fibrosis markers including N-cadherin, Collagen I, TGF-β1, α-SMA at different time points were detected by western blot assay. The results showed that compared with normal endometrial tissue, the overall expression levels of fibrosis markers in modeling 3d and 5d were slightly lower. While the above fibrosis markers expression significantly increased on the 7d, indicating that there was fibrous adhesion in the uterine cavity and IUA model was successfully constructed on this day, which can be used as the most suitable time for follow-up stem cell transplantation treatment (Fig. 3B).

### The combination of BMSCs with estrogen improves endometrial injure

**Fig. 4:**
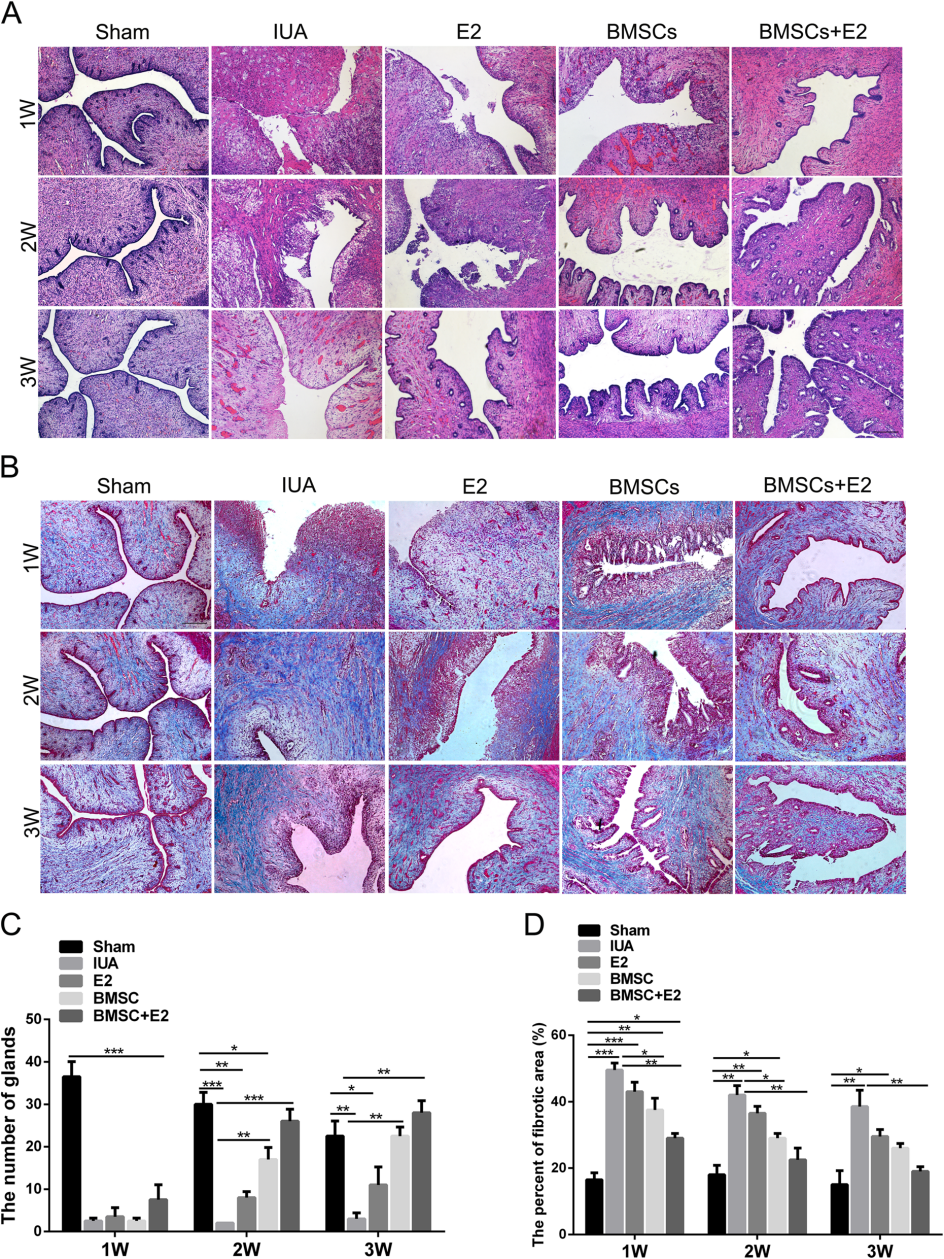
Evaluation of the effect of BMSCs combined with estrogen on endometrial morphological recovery. **A** HE staining was performed to detect the endometrial glands in each group at different time points (× 100, scale bar = 100 μm). **B** Masson staining was performed to detect intrauterine fibrosis changes in each group at different time points (× 100, scale bar = 100 μm). **C** Statistical results of changes in the number of endometrial glands. **D** Statistical results of intrauterine fibrosis changes after endometrial damage. ^*^p < 0.05, ^**^p < 0.01, ^***^p < 0.001

We collected the uterine tissues of each group at 1 week, 2 weeks and 3 weeks to observe a synergistic effect of BMSCs combined with estrogen therapy in promoting the endometrial morphology and function. According to H&E staining results, the shape of the uterine cavity was regular, a large number of single-layer columnar epithelium cells covered the uterus and gland cavities, the epithelial cells were structurally intact, and the interstitial glands were abundant and oval in the sham operation group. In the IUA model group, the structure of the uterine cavity was destroyed and the number of glands were significantly reduced with connective tissue fragments. However, after BMSCs or estrogen treatment, the number of endometrial glands increased to varying degrees with the prolongation of the treatment time, and the above results were most noteworthy in BMSCs combined with estrogen treatment group (Fig. 4A and C). Masson staining showed that there was almost no blue collagen deposition in the endometrial stroma in the sham operation group. In contrast, a large number of blue collagen fibers formed and accumulated in the uterine cavity, resulting in the formation of obvious adhesion bands in the IUA model group. In other treatment groups (BMSC, E2, and BMSC + E2), the proportion of fibrosis area decreased gradually and collagen fibers gradually arranged in order with the extension of treatment time, especially in the group that received the BMSCs in combination with estrogen (Fig. 4B and D). Taken together, these results suggested that BMSCs combined with estrogen significantly improved endometrial injure in rabbit IUA model.

### BMSCs combined with estrogen reduces endometrial fibrosis

**Fig. 5:**
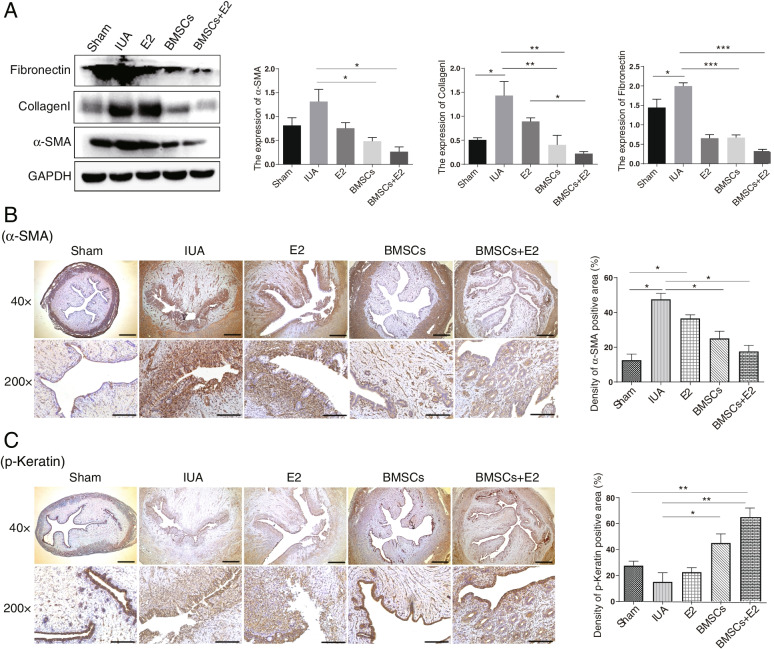
Endometrial fibrosis changes after BMSCs combined with estrogen treatment. **A** Western blot was used to detect the expression of fibrotic markers in endometrial tissues of each group. **B** Immunohistochemical staining to identify the expression of α-SMA in different treatment groups. **C** Immunohistochemical staini was used to detect p-Keratin expression in different treatment groups (× 40, scale bar = 200 μm; × 200, scale bar = 50 μm). ^*^p < 0.05, ^**^p < 0.01

Western blot was used to detect the expression levels of fibrosis markers including Fibronectin, Collagen I and α-SMA. Results showed that compared with the sham operation group, the above mentioned proteins expression increased in the IUA group, but decreased in all treatment groups, and the reduction was the most obvious in the BMSCs + E2 group (Fig. 5A). Meanwhile, IHC staining was also used to investigate α-SMA expression. We found that the expression of α-SMA was significantly increased and the degree of fibrosis was aggravated in the IUA group, while decreased in the E2 group and BMSCs group, especially in the combined therapy group (Fig. 5B). In addition, we also evaluated p-keratin expression, a glandular epithelium marker. As shown in Fig. 5C, the positive staining areas of normal endometrium are brown-yellow color, but less positive staining were observed in IUA group. Interestingly, a small amount of brown-yellow particles were observed in the endometrial epithelium of E2 treatment group or BMSCs treatment group. And the positive staining in the BMSCs + E2 combination group was significantly increased due to the newly formation of large amount of luminal epithelium and glandular epithelium (Fig. 5C). Taken together, the above results indicate that the combination treatment of BMSCs and E2 can promote endometrial repair by reducing endometrial fibrosis.

### BMSCs combined with estrogen inhibits EMT occurrence

**Fig. 6:**
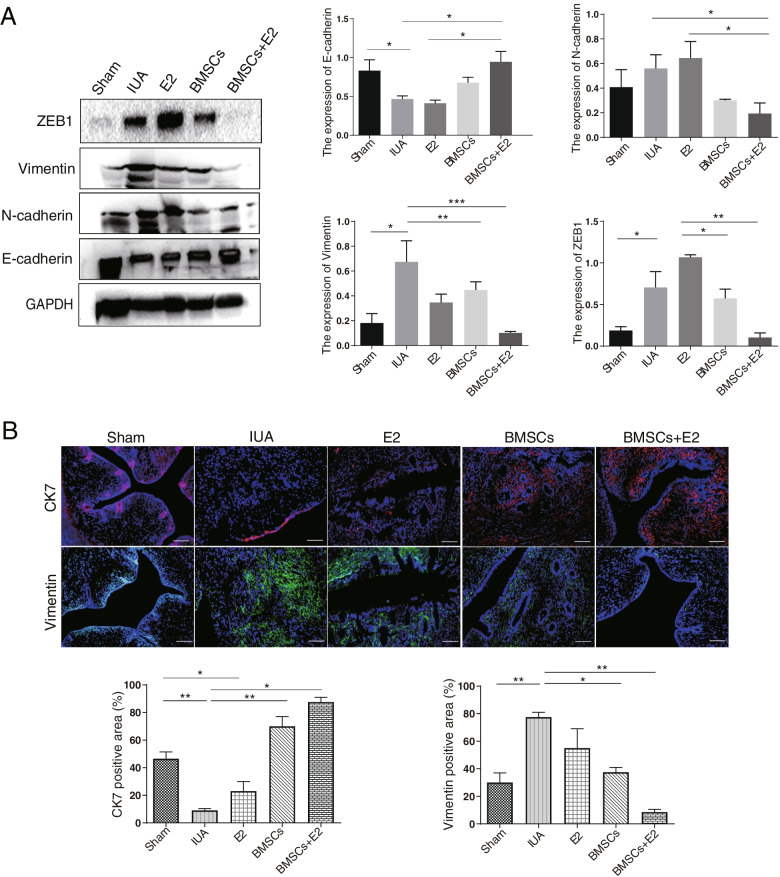
Effect of BMSCs combined with estrogen therapy on EMT. **A** Western blot was used to detect EMT markers expression in endometrial tissues. **B** Immunofluorescence staining to detect the expression of CK7 and Vimentin (× 200, scale bar = 50 μm). ^*^p < 0.05, ^**^p < 0.01

To explore the underlying mechanism of BMSCs combined with estrogen therapy in endometrial repair, the expression of EMT markers was explored. As expected, the results indicated that the expression of N-cadherin, Vimentin and ZEB1 was significantly increased, while E-cadherin expression was decreased in the IUA group. However, the changes of the above molecular proteins were completely opposite in the BMSCs + E2 group (Fig. 6A). To further validate the effect of the combination therapy on EMT, immunofluorescent staining was performed to analyze the abundance and distribution of CK7 and Vimentin in uterine cavity. The results showed that CK7, an epithelial-specific marker, is expressed in normal endometrial luminal and glandular epithelium, Vimentin is specifically expressed in the normal endometrial stroma. But CK7 expression was almost absent and Vimentin expression was significantly elevated in the IUA group. Interestingly, the abundance of CK7 was increased remarkably in the combination treatment group and mainly localized in the glands and surrounding tissues, while Vimentin expression was decreased and primarily located in the endometrial stroma (Fig. 6B). These results indicate that BMSC transplantation combined with estrogen therapy can promote epithelial cell expression and inhibit mesenchymal cell expression, suggesting that combined therapy can further promote endometrial repair by inhibiting the EMT occurrence.

### The effect of BMSC combined with estrogen treatment on the protein expression of Wnt/β-catenin pathway

**Fig. 7:**
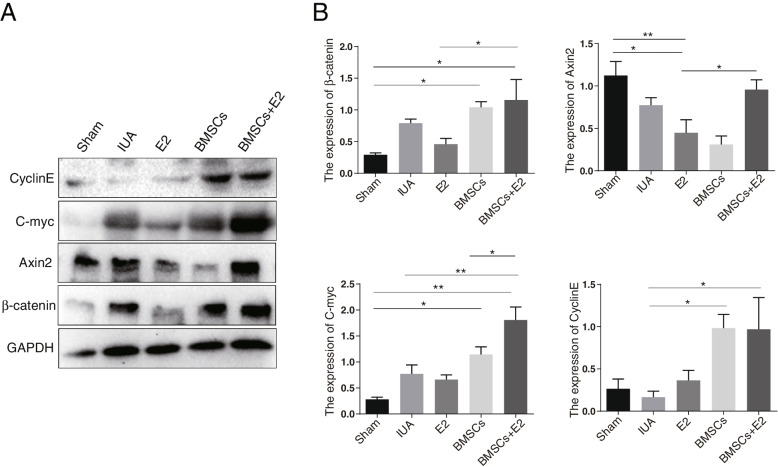
The expression of core molecules of Wnt signaling pathway in rabbit endometrium. **A** Western blot detected the protein expression of CyclinE, C-myc, Axin2 and β-catenin in different groups. **B** Quantitative analysis for CyclinE, C-myc, Axin2 and β-catenin expression. ^*^p < 0.05, ^**^p < 0.01

To determine whether the Wnt/β-catenin pathway was involved in the process of BMSCs combined with estrogen to inhibit EMT and fibrosis, western blot was performed to detect the protein expression levels of CyclinE, C-myc, Axin2 and β-catenin. The results demonstrated that β-catenin and C-myc expression were increased in the IUA model group. However, the expression of above-mentioned proteins was basically unchanged in the estrogen treatment group, increased in the BMSC treatment group, and the increase was even greater in BMSC + E2 group (Fig. 7A and B). The above results indicated that BMSCs combined with estrogen therapy inhibited EMT and endometrial fibrosis, which partly through activating the Wnt/β-catenin signaling pathway.

## Discussion

Common treatment options for IUA include hysteroscopic adhesiolysis and hormone therapy. Despite the cure rate of IUA has improved, the recurrence rate still high and the treatment remains a challenge [22]. It has been reported that the regeneration and repair of endometrium depend on the proliferation and differentiation of the basal layer stem cells [23]. Recently, much attention has been focus on the treatment of IUA with mesenchymal stem cell (MSC) theory [24] and improving cell survival ability and utilization rate is the key to strengthen stem cell-based endometrial repair. Increasing studies have shown that BMSCs were famous for their advantages of proliferative ability and multi-differentiation potential, and stem cells could migrate to the injured area of the uterine cavity to promote endometrial regeneration [25]. PKH26 is a simple and effective tracer labeling technique in vivo that can track the migration and distribution of MSC. In this study, we have successfully conducted PKH26-labeled BMSCs transplantation into uterine cavity of IUA models and found that BMSCs mainly located in the endometrial glands and surrounding matrix, indicating that BMSCs may differentiate into epithelial cells to promote the repair of damaged endometrium. In addition, BMSCs decreased gradually with the extension of transplantation time. However, it is interesting to note that the effect of BMSCs on endometrial regeneration was more significant at 2 weeks and 3 weeks after orthotopic transplantation than at 1 week. Therefore, these results indicate that BMSCs may promote endometrial repair through secreting other factors temporarily, which was consistent with the findings reported in previous studies [13].

More and more studies have shown that BMSCs combined with other methods or biomaterials can exert better therapeutic effect. Estrogen has been proved to be effective in preventing postoperative re-adhesion, restoring menstruation, and improving endometrial thickness in patients with injured endometrium [26]. Exogenous estrogen plays an important role in promoting the differentiation of endometrial basal stem cells during IUA treatment [27]. Our previous study have demonstrated that estrogen may prevent the occurrence and development of IUA by inhibiting the TGF-β1 induced EMT and activating the Wnt/β-catenin pathway [28]. In this study, administration of BMSCs and estrogen obviously increased the number of endometrial glands and decreased the degree of endometrial fibrosis in IUA rabbits model, and the combination strategy comprising the best results than BMSCs or estrogen alone, which was consistent with the results of previous report [15]. BMSCs transplantation with estrogen as the carrier can be applied to treat endometrial injury in rabbits. Estrogen plays an important role in improving utilization rates of BMSCs in the repair of endometrium, most likely by promoting the migration and differentiation of BMSCs into endometrial epithelial cells. Moreover, estrogen may provide a suitable environment for BMSCs to secrete related factors.

The most important pathological features of IUA is intrauterine fibrosis [29, 30]. Fibronectin, Collagen I and α-SMA are specific markers of fibrogenesis and closely related to cell fibrosis. In the current study, the expression of these markers were significantly decreased in the estrogen/BMSCs treated group, suggesting that the combination both of them inhibited fibrosis progression and contributed to the repair of endometrial epithelium. EMT is always accompanied by fibrogenesis during IUA [31]. Hence, we also explored the synergistic effect of BMSCs combined with estrogen on EMT, and results indicated that the expression of epithelial markers was increased and interstitial markers was decreased in the combination therapy group, indicating that BMSCs/estrogen can promote re-epithelization through reverse EMT occurrence. Previous studies have demonstrated that Wnt/β-catenin signaling pathway is closely related to EMT in endometrial fibrosis [32, 33]. So we studied the functions and mechanisms of Wnt signaling in IUA treatment with BMSCs transplantation. In our rabbit model of IUA, the expression of key proteins in Wnt signaling was detected in the uterus tissues. The results indicated that Wnt/β-catenin signaling activation in the BMSCs and BMSCs + E2 groups, suggesting that combination strategy has the best therapeutic effect and synergistically inhibiting EMT partly through activating the Wnt/β-catenin pathway.

## Conclusions

In conclusion, the present study confirmed that estrogen supports the differentiation of BMSCs into endometrial epithelial cells and thus enhancing the therapeutic effect of transplanted cells in IUA. The synergistic effect of BMSCs combined with estrogen was achieved by inhibiting EMT and endometrial fibrosis, and this combined effect was enhanced in partly through activation of the Wnt/β-catenin signaling pathway. In the future, estrogen combined with BMSCs transplantation is a promising strategy, which provides a reliable alternative for the clinical treatment of IUA.

## Supplementary Information

**Additional file 1.**

**Additional file 2.**

**Additional file 3.**

**Additional file 4.**

**Additional file 5.**

## Data Availability

All data generated or analyzed during this study are included in this published article.

## Abbreviations

BMSCs: Bone marrow mesenchymal stem cells
MSCs: Mesenchymal stem cells
IUA: Intrauterine adhesion
E2: Estrogen
EMT: Epithelial-mesenchymal transition
ADSCs: Autologous adipose-derived stem cells
