# Correction: Bone marrow mesenchymal stem cells combined with estrogen synergistically promote endometrial regeneration and reverse EMT via Wnt/β-catenin signaling pathway

**DOI:** 10.1186/s12958-022-01047-5

**Published:** 2022-12-20

**Authors:** Liwei Yuan, Jia Cao, Mingyue Hu, Dabao Xu, Yan Li, Shiyun Zhao, Juanjuan Yuan, Huixing Zhang, Yani Huang, He Jin, Meixia Chen, Dan Liu

**Affiliations:** 1 Department of Gynecology, General Hospital of Ningxia Medical University, Yinchuan, Ningxia China; 2 College of Clinical Medicine, Ningxia Medical University, Yinchuan, Ningxia China; 3 Department of Beijing National Biochip Research Center Sub-Center in Ningxia, General Hospital of Ningxia Medical University, Yinchuan, Ningxia China; 4 Department of Gynecology, Third Xiangya Hospital of Central South University, Changsha, Hunan China; 5 Key Laboratory of Ministry of Education for Fertility Preservation and Maintenance, Ningxia Medical University, Yinchuan, Ningxia China

**Correction: Reprod Biol Endocrinol 20, 121 (2022)**

**https://doi.org/10.1186/s12958-022-00988-1**

Following publication of the original article [1], the authors identified an error in Figs. 3 and 4. The correct figures are given below.

**Fig. 3:**
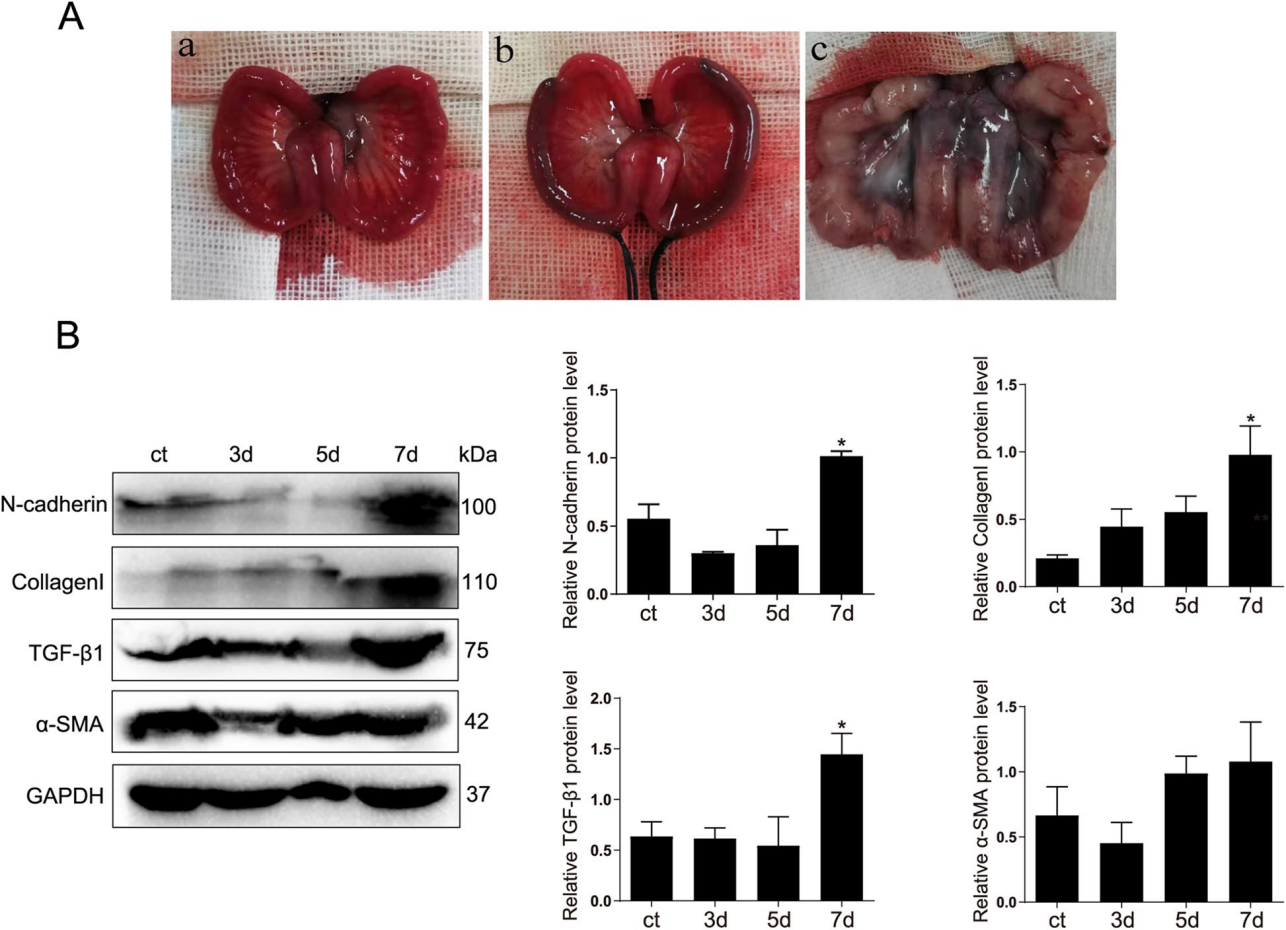
Quantitative assessment of BMSCs transplantation time. **A** (a): The normal uterine morphology of a rabbit; (b): Morphology of rabbit uterus after mechanical and infectious double injury; (c): Morphology after one week of injury). **B** Western blot was used to detect the protein expression of fibrosis markers (N-cadherin, Collagen I, TGF-β1, α-SMA) at different time points. ^*^p < 0.05. The explanation for the cropping of gels and blots in the experiment is as follows: the same proteins were separated by electrophoresis and transferred to PVDF membrane. According to the different molecular weight of the target antibody, the protein on a PVDF membrane was cropped horizontally. Then, the corresponding antibody was incubated and exposed, and the position of antibody was indicated by protein marker to ensure the exclusion of interference from non-specific antibody magazines

**Fig. 4:**
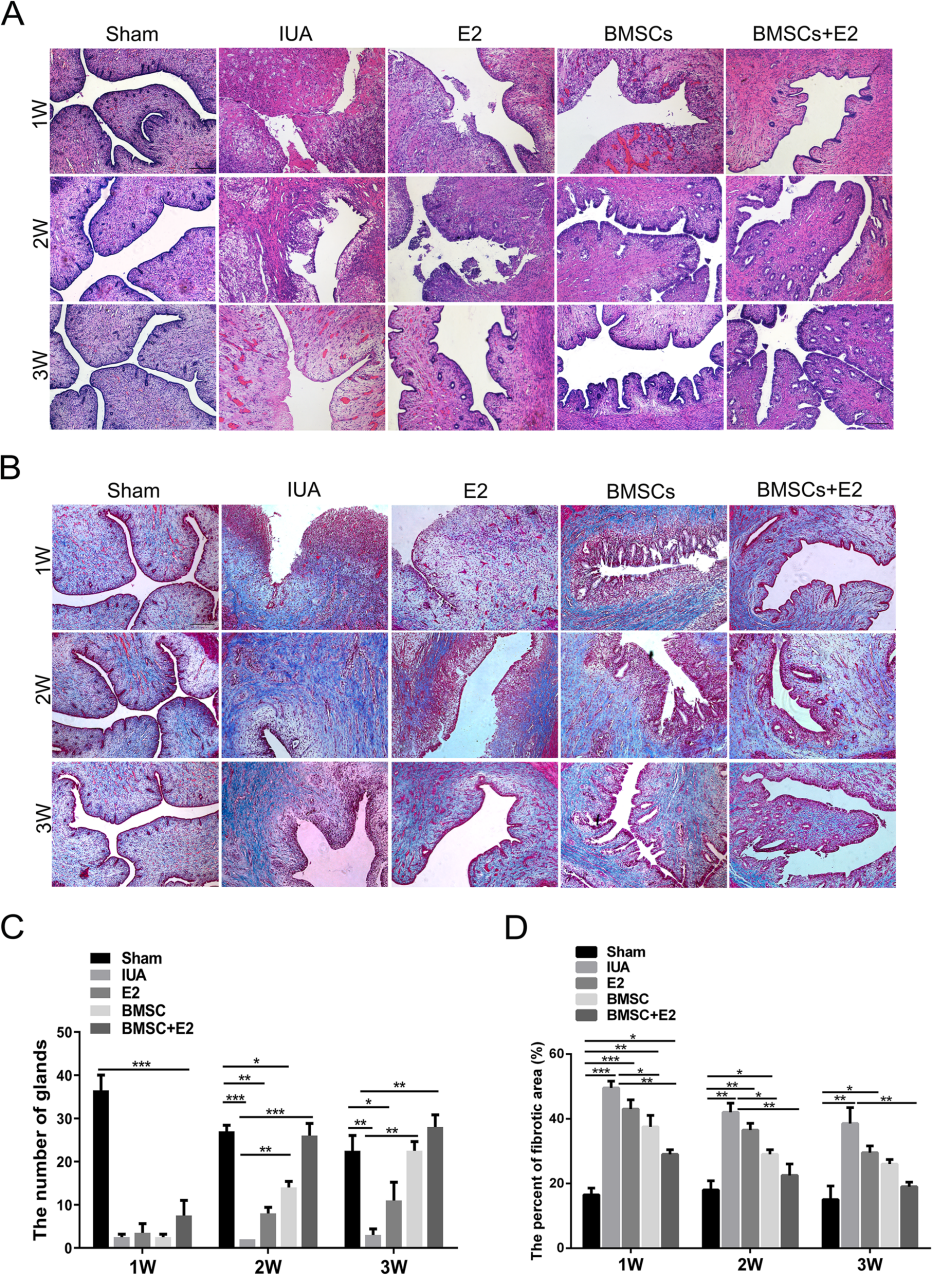
Evaluation of the effect of BMSCs combined with estrogen on endometrial morphological recovery. **A** HE staining was performed to detect the endometrial glands in each group at different time points (× 100, scale bar = 100 μm). **B** Masson staining was performed to detect intrauterine fibrosis changes in each group at different time points (× 100, scale bar = 100 μm). **C** Statistical results of changes in the number of endometrial glands. **D** Statistical results of intrauterine fibrosis changes after endometrial damage. ^*^p < 0.05, ^**^p < 0.01, ^***^p < 0.001

The original article [1] has been updated.
