# Supplementary figures and images for: Bone marrow mesenchymal stem cells combined with estrogen synergistically promote endometrial regeneration and reverse EMT via Wnt/β-catenin signaling pathway
Source: Reprod Biol Endocrinol. 2022 Aug 15;20:121. doi: 10.1186/s12958-022-00988-1 (PMC9377128; doi:10.1186/s12958-022-00988-1)

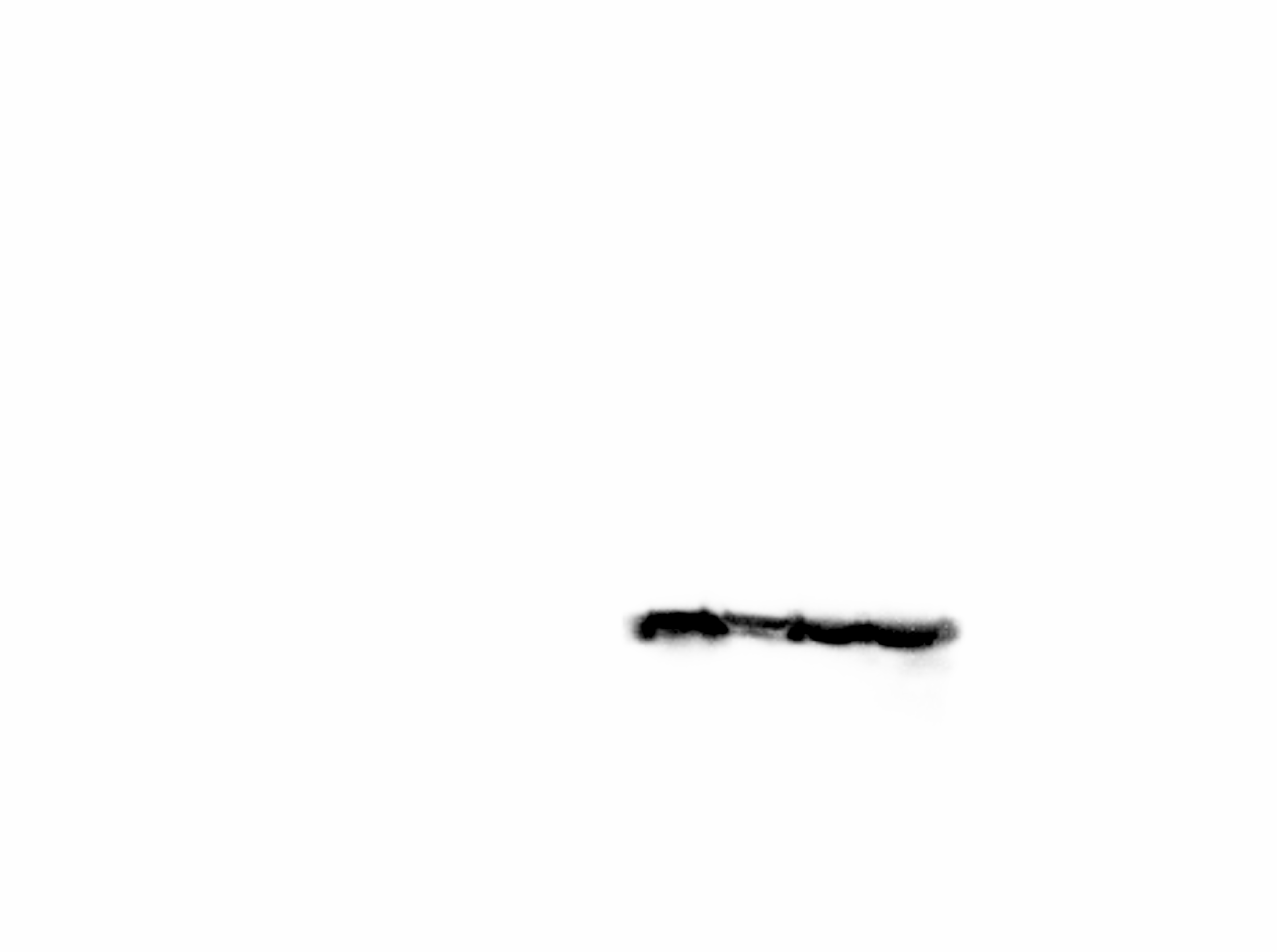

Supplement: Supplementary file 1 — Additional file 1. [file 12958_2022_988_MOESM1_ESM.zip › Fig. 3BaSMA.tif]

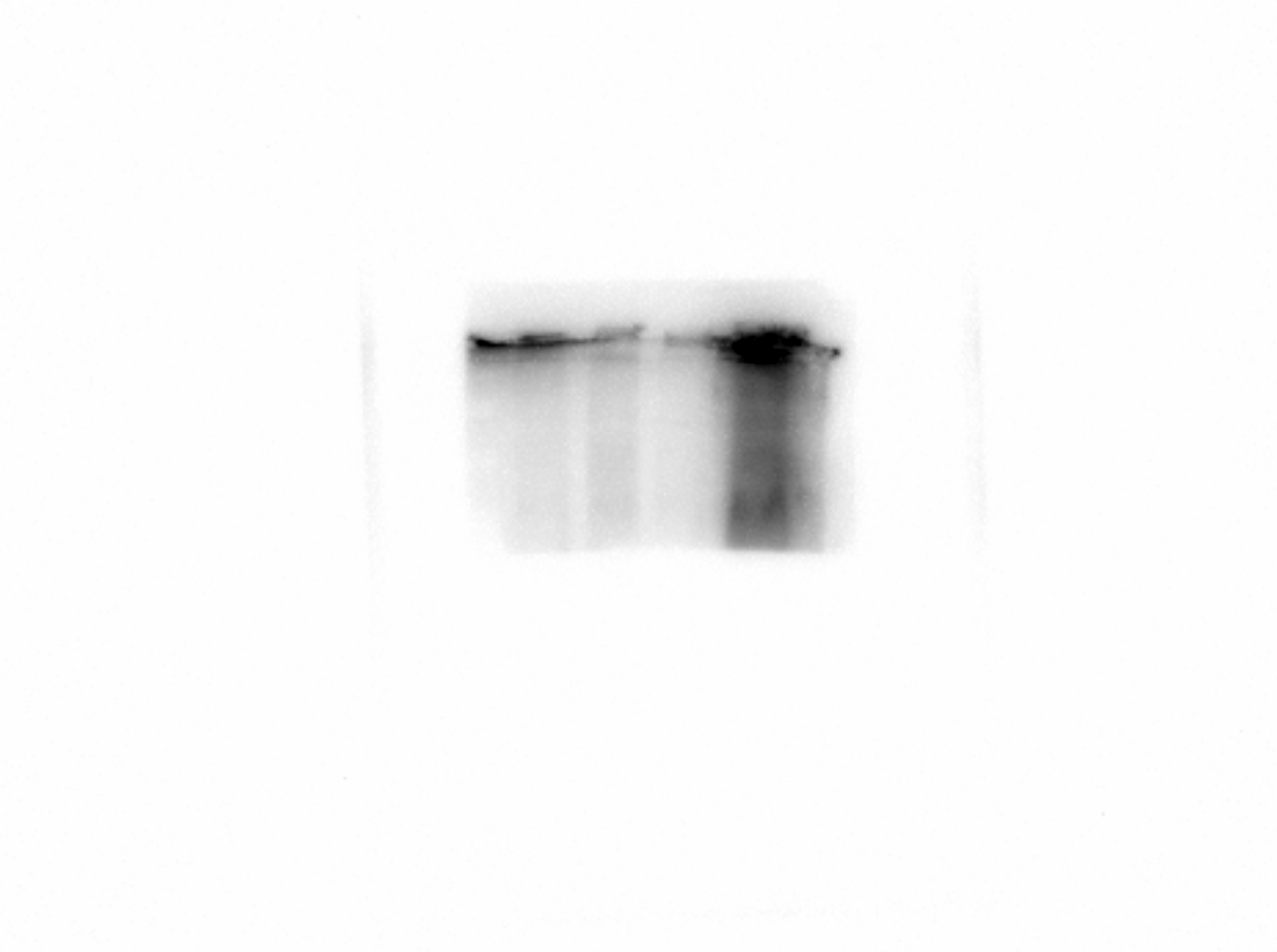

Supplement: Supplementary file 1 — Additional file 1. [file 12958_2022_988_MOESM1_ESM.zip › Fig. 3Bcadherin.tif]

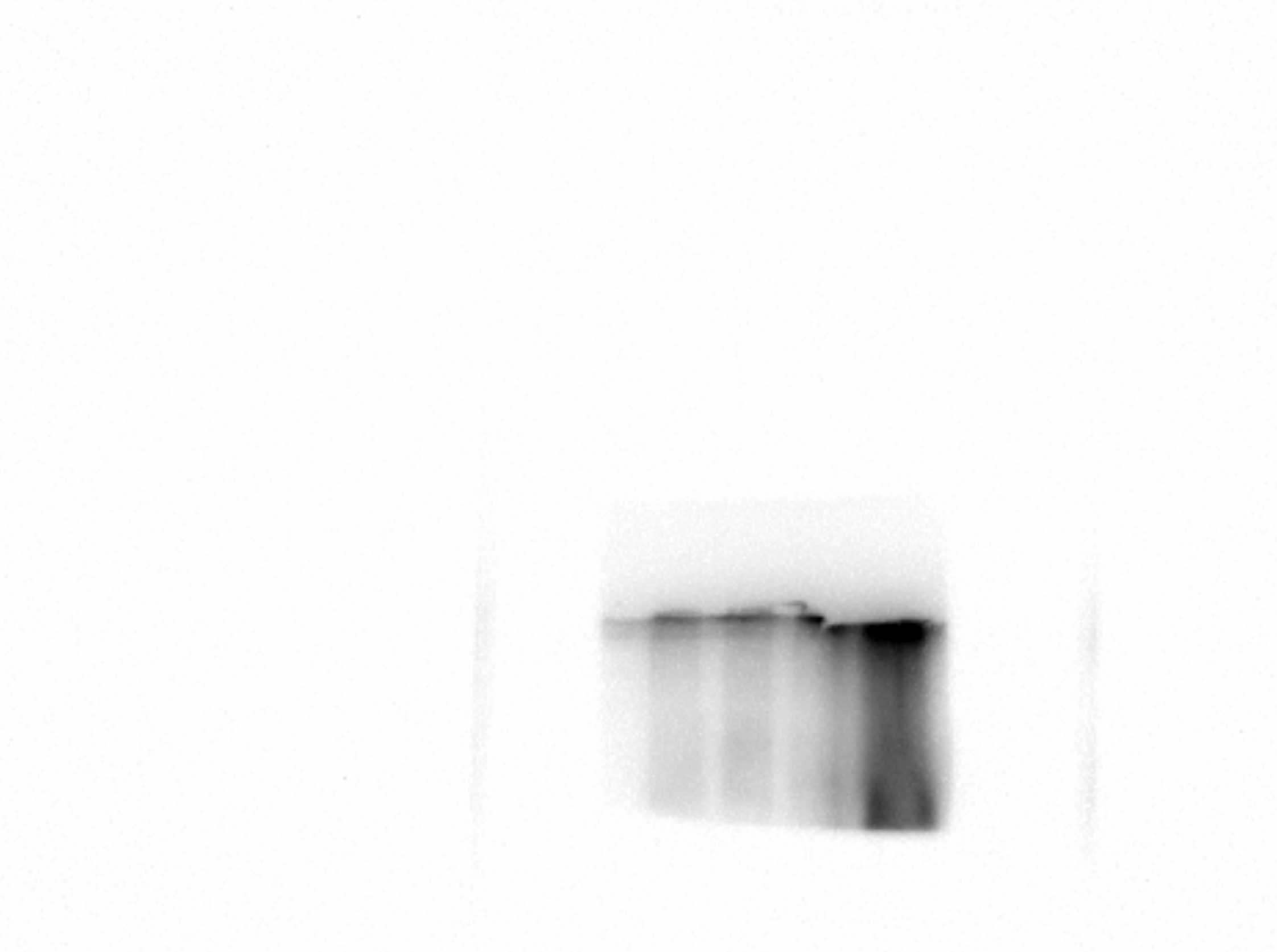

Supplement: Supplementary file 1 — Additional file 1. [file 12958_2022_988_MOESM1_ESM.zip › Fig. 3BCollagenI.tif]

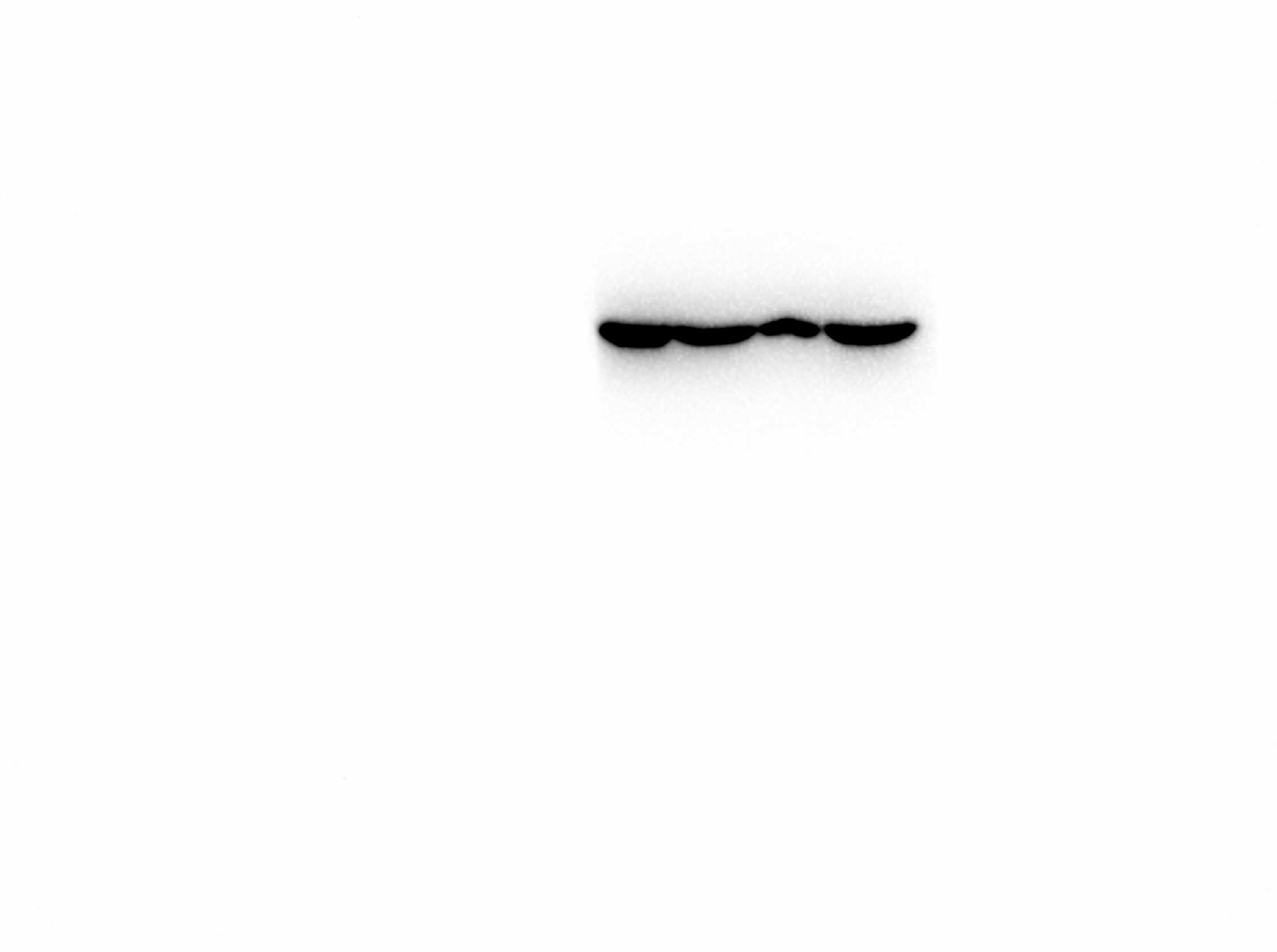

Supplement: Supplementary file 1 — Additional file 1. [file 12958_2022_988_MOESM1_ESM.zip › Fig. 3BGAPDH.tif]

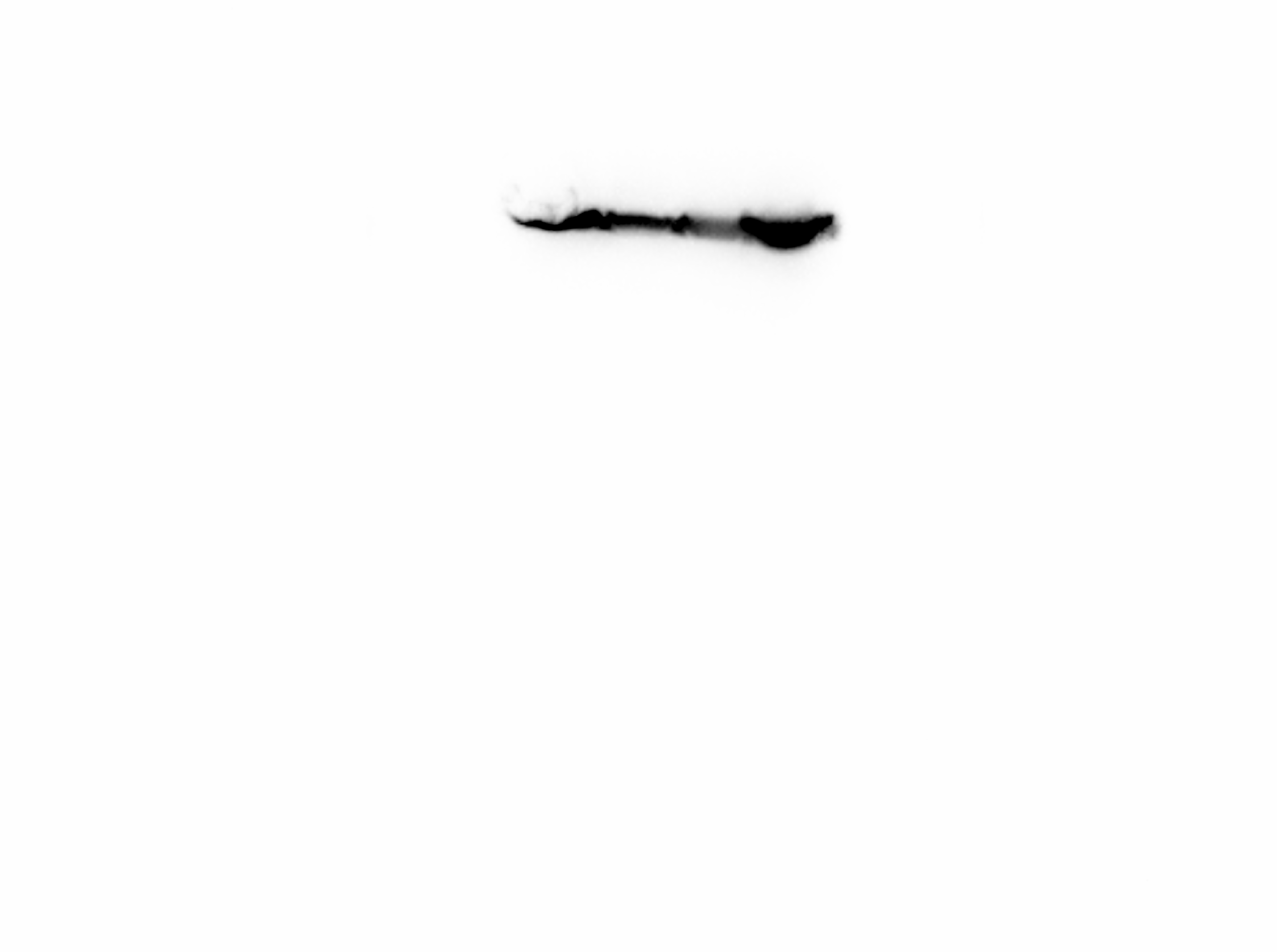

Supplement: Supplementary file 1 — Additional file 1. [file 12958_2022_988_MOESM1_ESM.zip › Fig. 3BTGF1.tif]

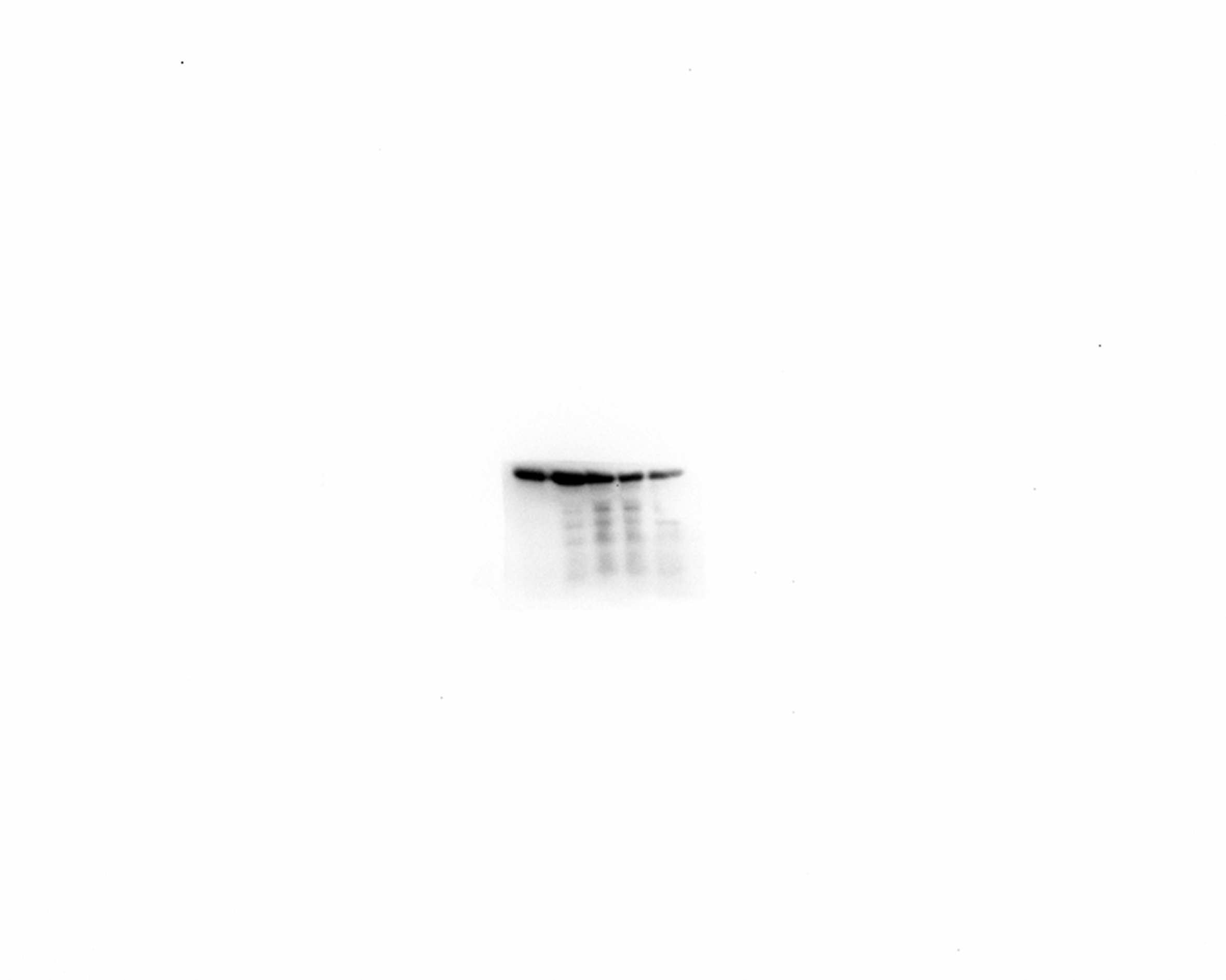

Supplement: Supplementary file 2 — Additional file 2. [file 12958_2022_988_MOESM2_ESM.zip › Fig. 5AaSMA.jpg]

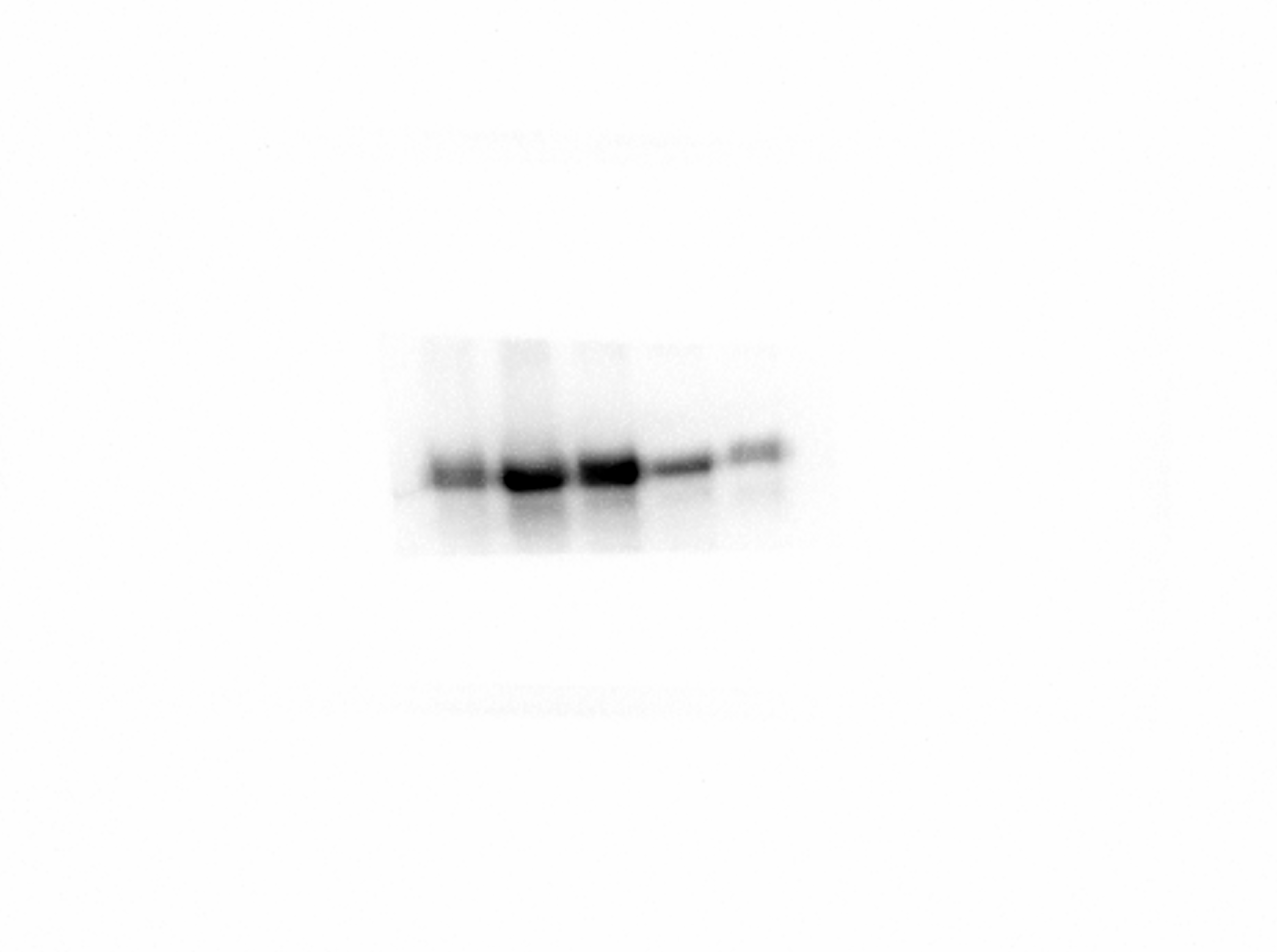

Supplement: Supplementary file 2 — Additional file 2. [file 12958_2022_988_MOESM2_ESM.zip › Fig. 5ACollagenI.tif]

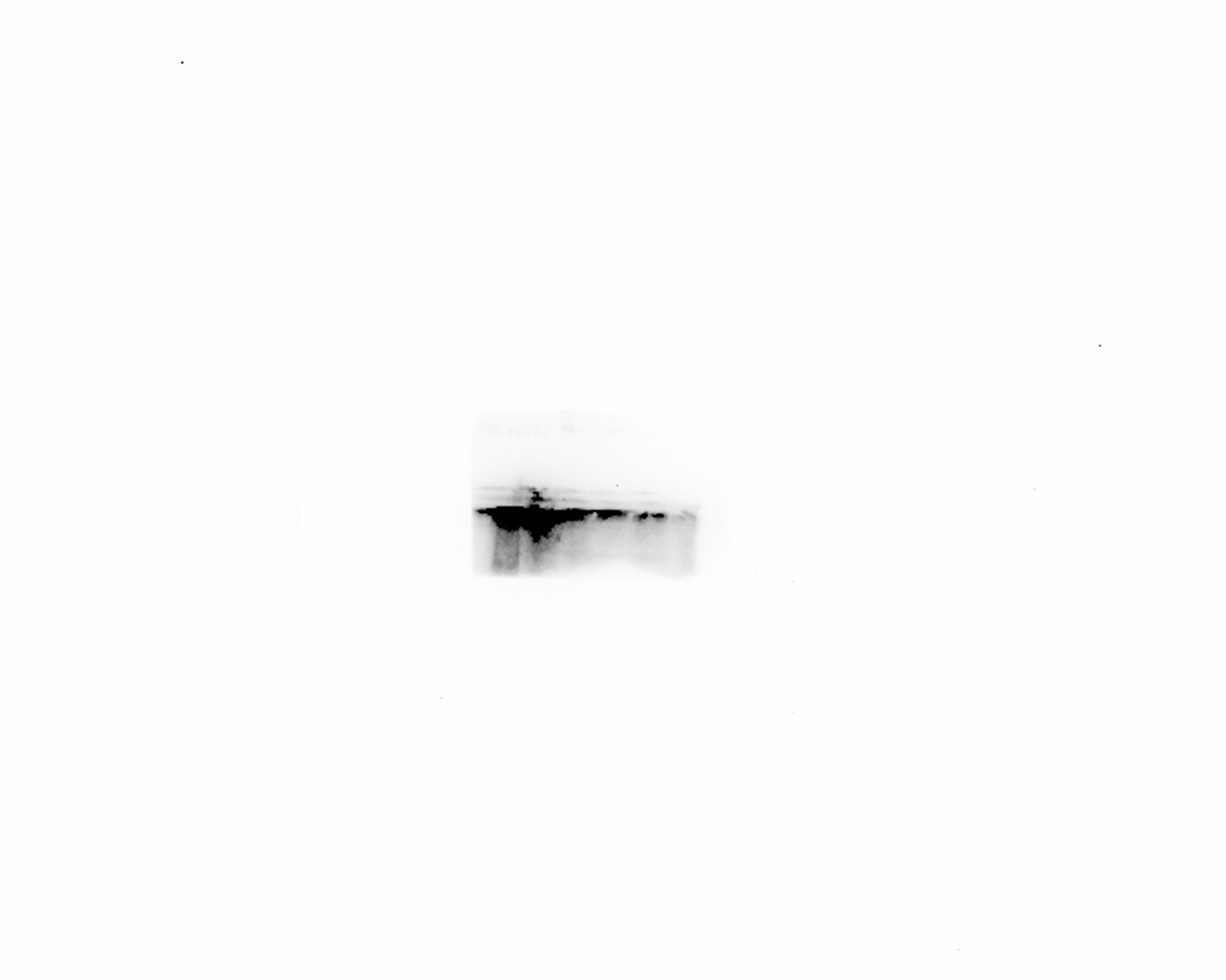

Supplement: Supplementary file 2 — Additional file 2. [file 12958_2022_988_MOESM2_ESM.zip › Fig. 5AFibronectin.jpg]

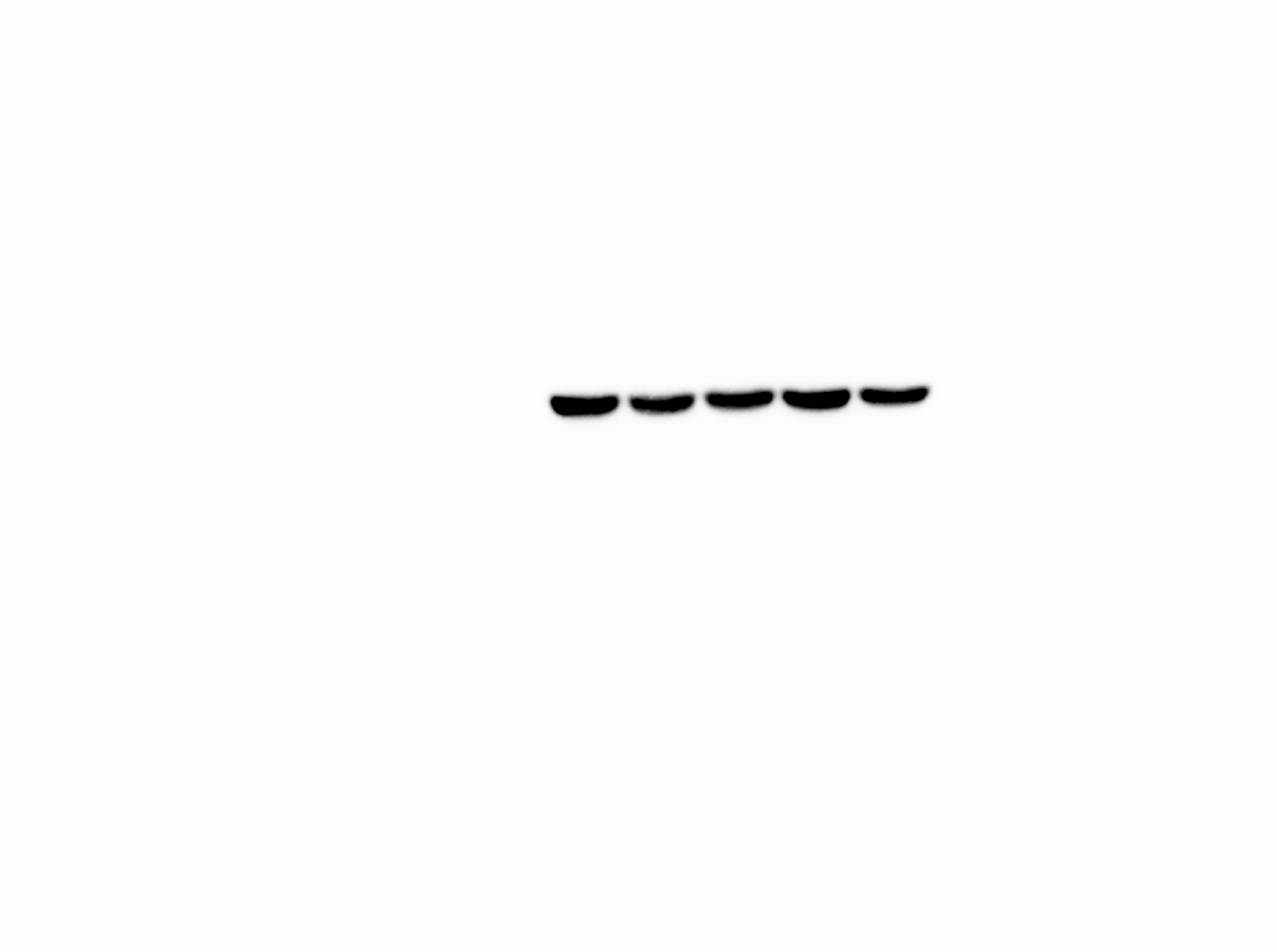

Supplement: Supplementary file 2 — Additional file 2. [file 12958_2022_988_MOESM2_ESM.zip › Fig. 5AGAPDH.tif]

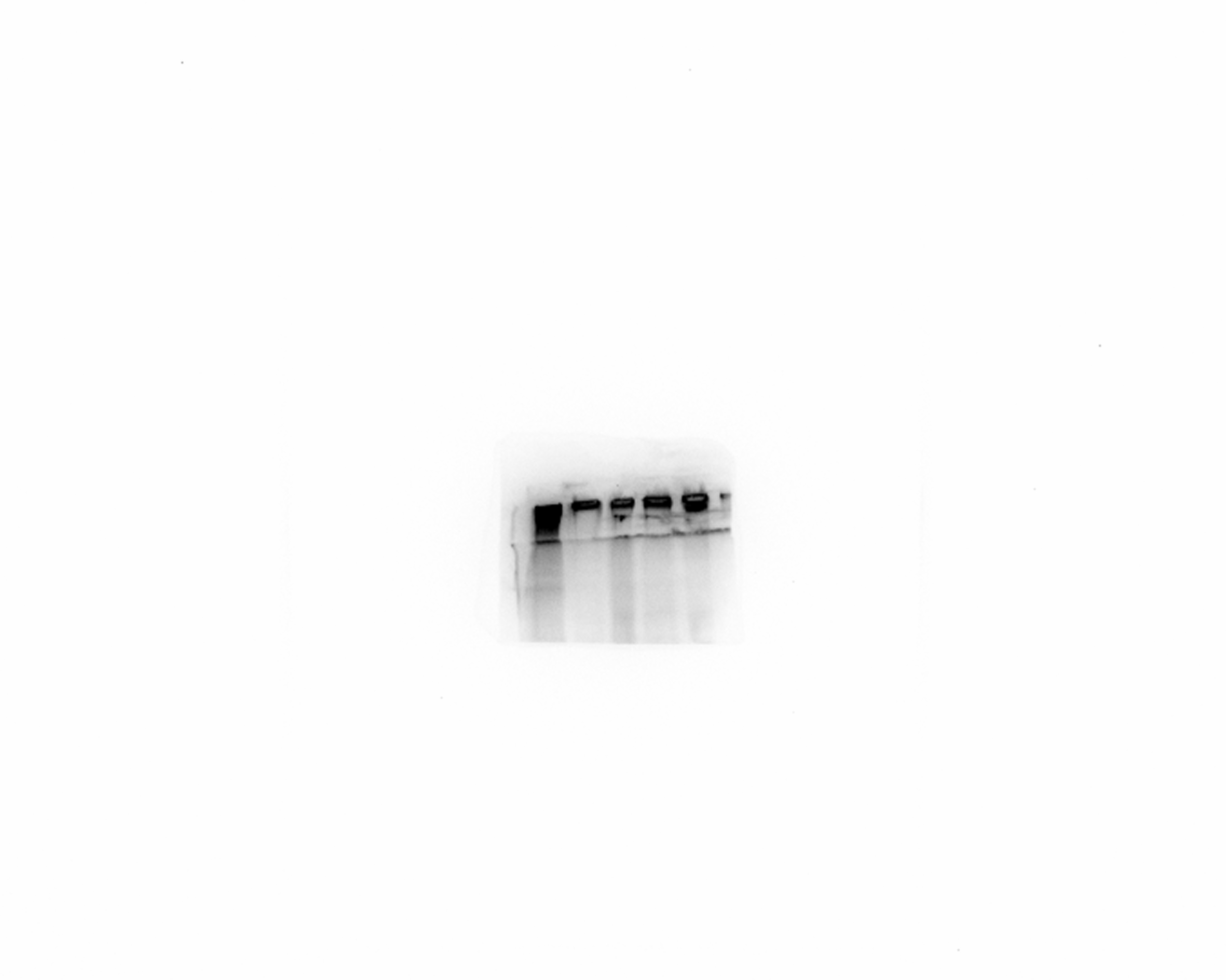

Supplement: Supplementary file 3 — Additional file 3. [file 12958_2022_988_MOESM3_ESM.zip › Fig. 6A--E-cadherin.jpg]

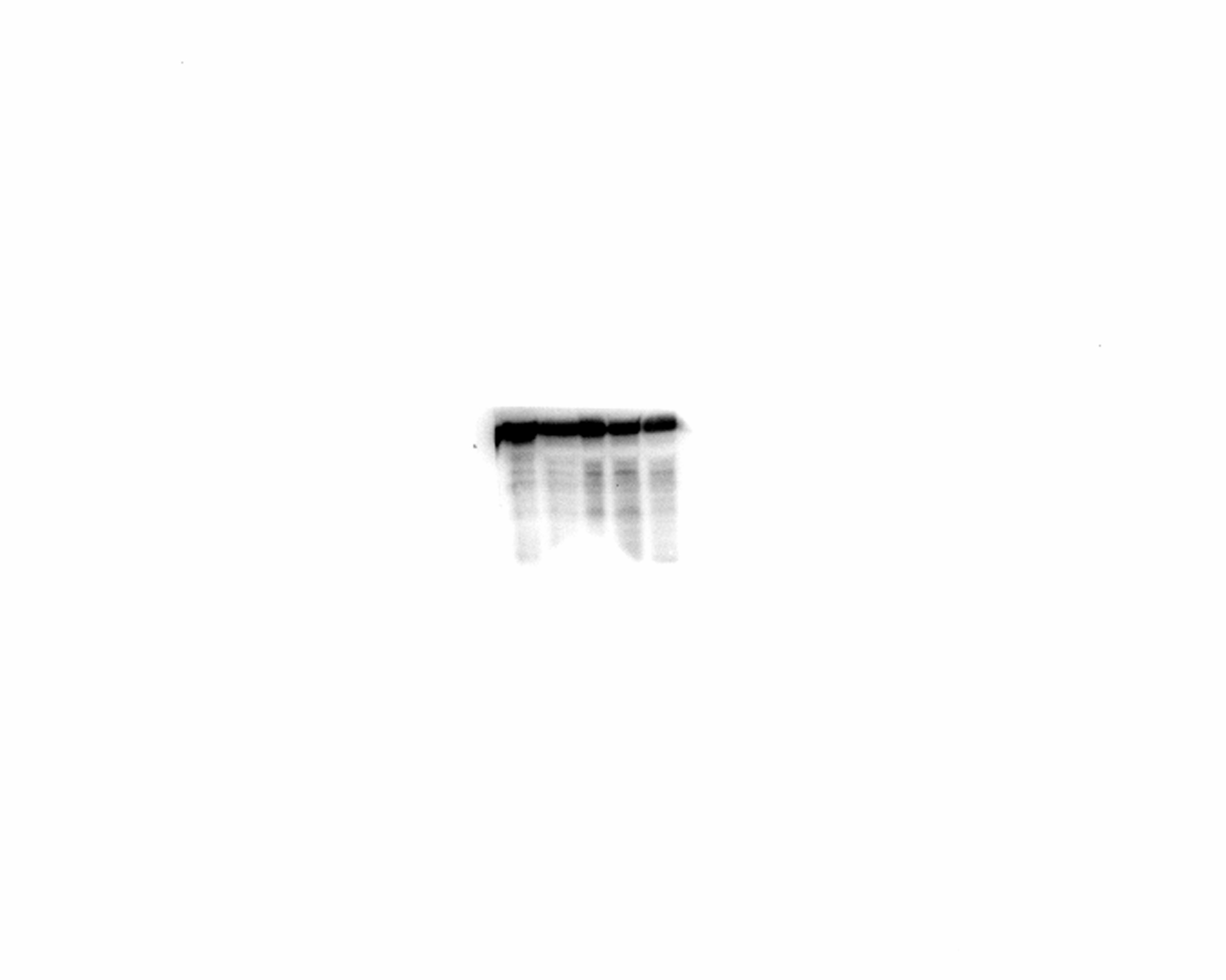

Supplement: Supplementary file 3 — Additional file 3. [file 12958_2022_988_MOESM3_ESM.zip › Fig. 6A--GAPDH.jpg]

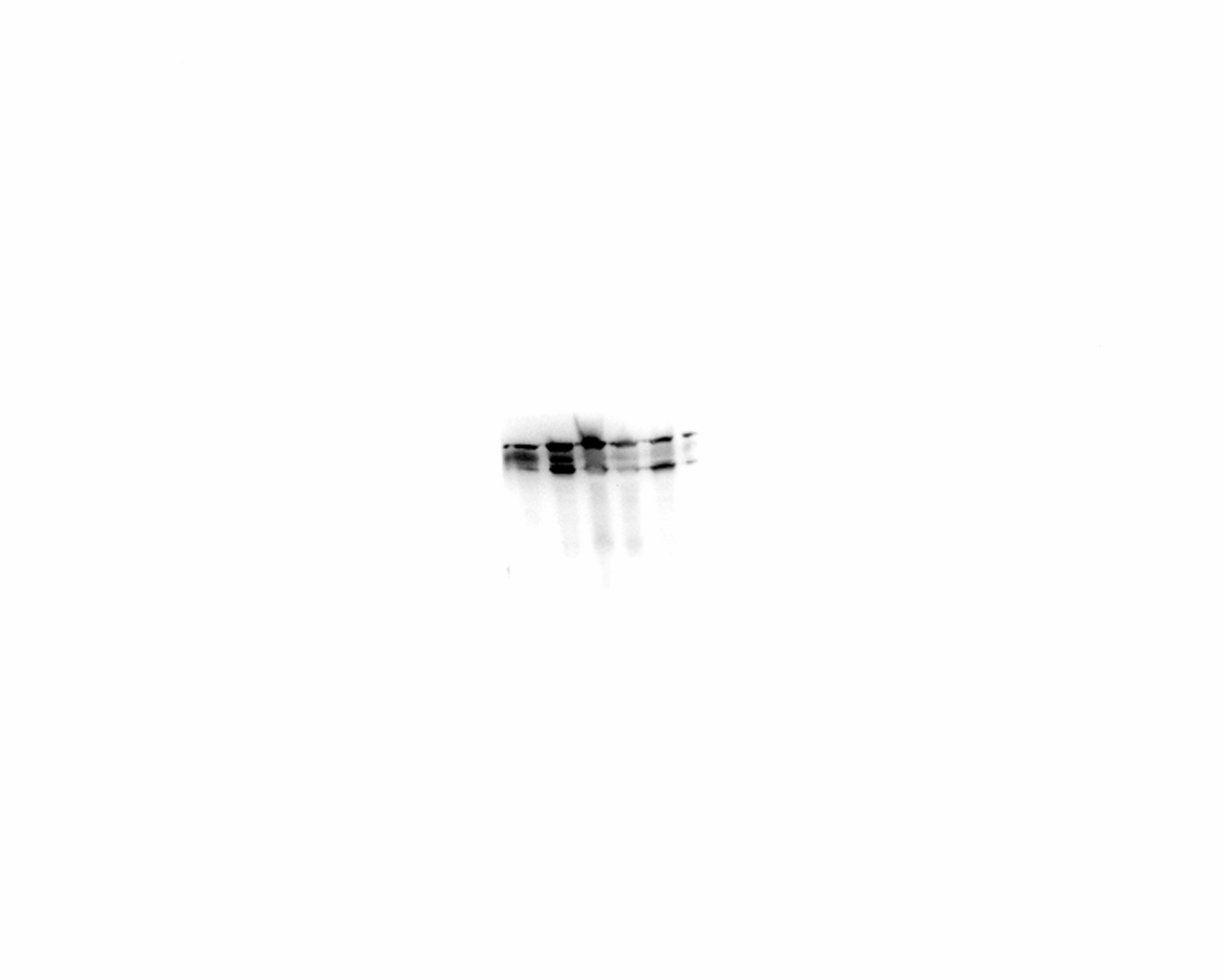

Supplement: Supplementary file 3 — Additional file 3. [file 12958_2022_988_MOESM3_ESM.zip › Fig. 6A--N-cadherin.jpg]

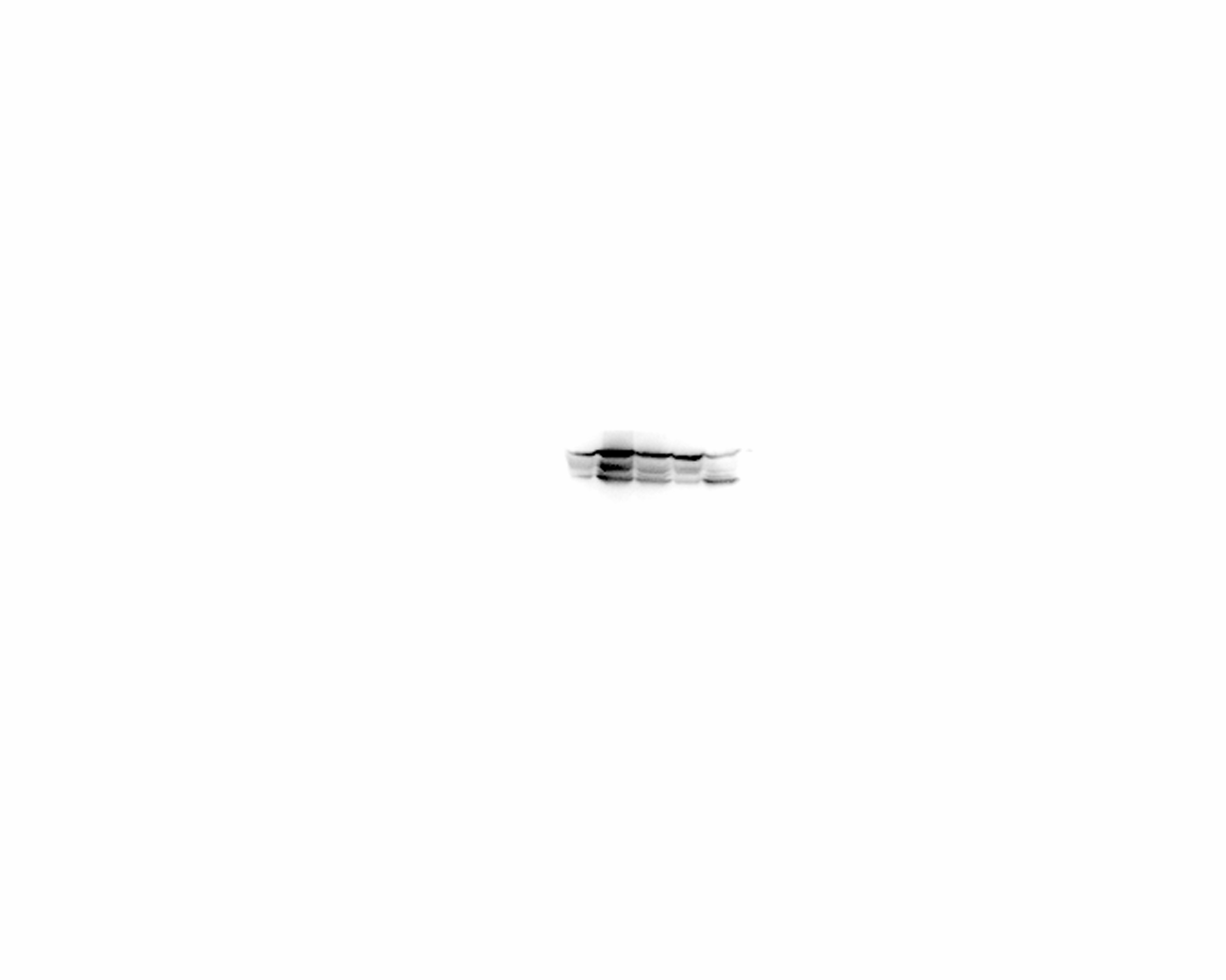

Supplement: Supplementary file 3 — Additional file 3. [file 12958_2022_988_MOESM3_ESM.zip › Fig. 6A--Vimentin.jpg]

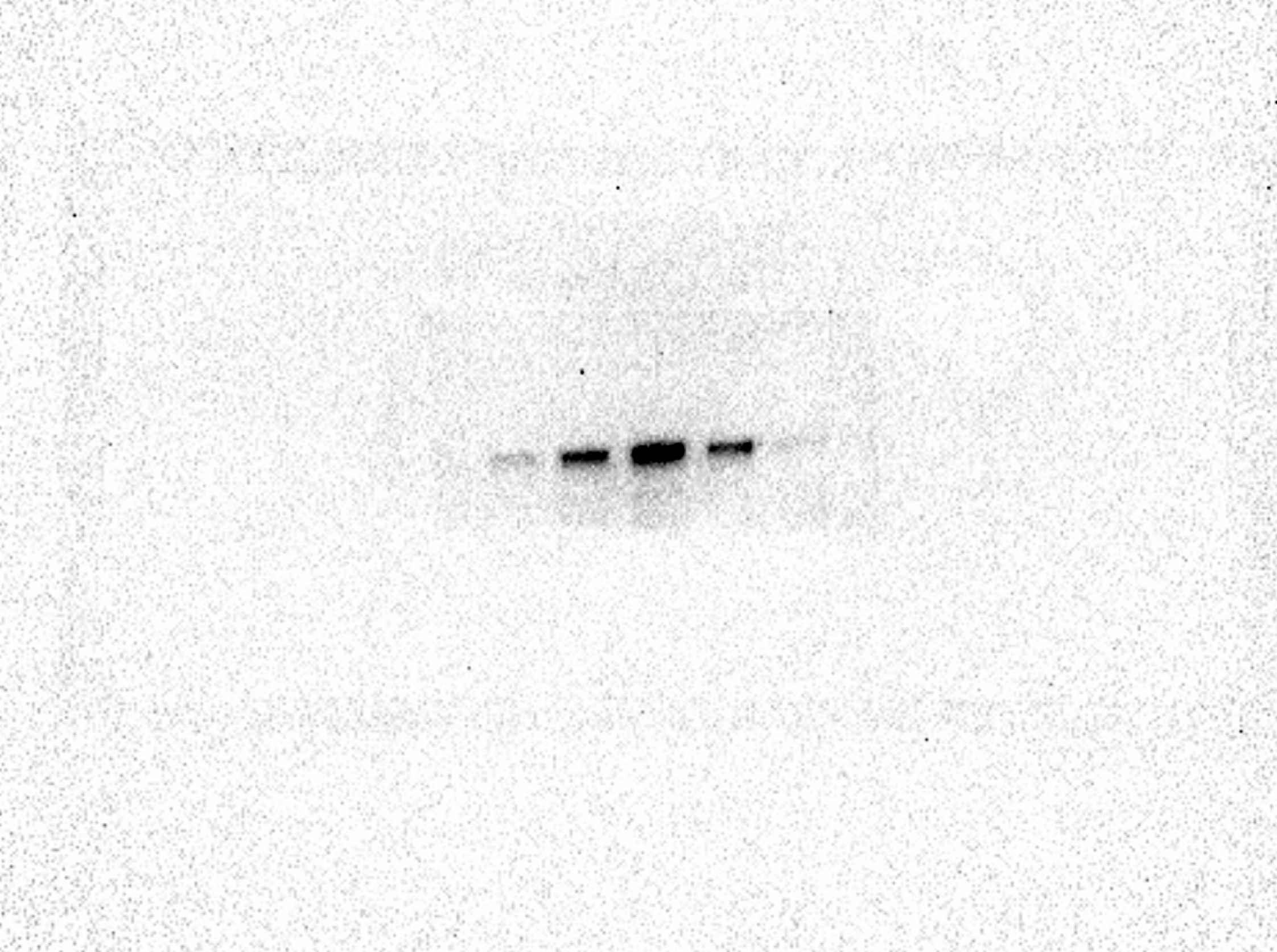

Supplement: Supplementary file 3 — Additional file 3. [file 12958_2022_988_MOESM3_ESM.zip › Fig. 6A--ZEB1.tif]

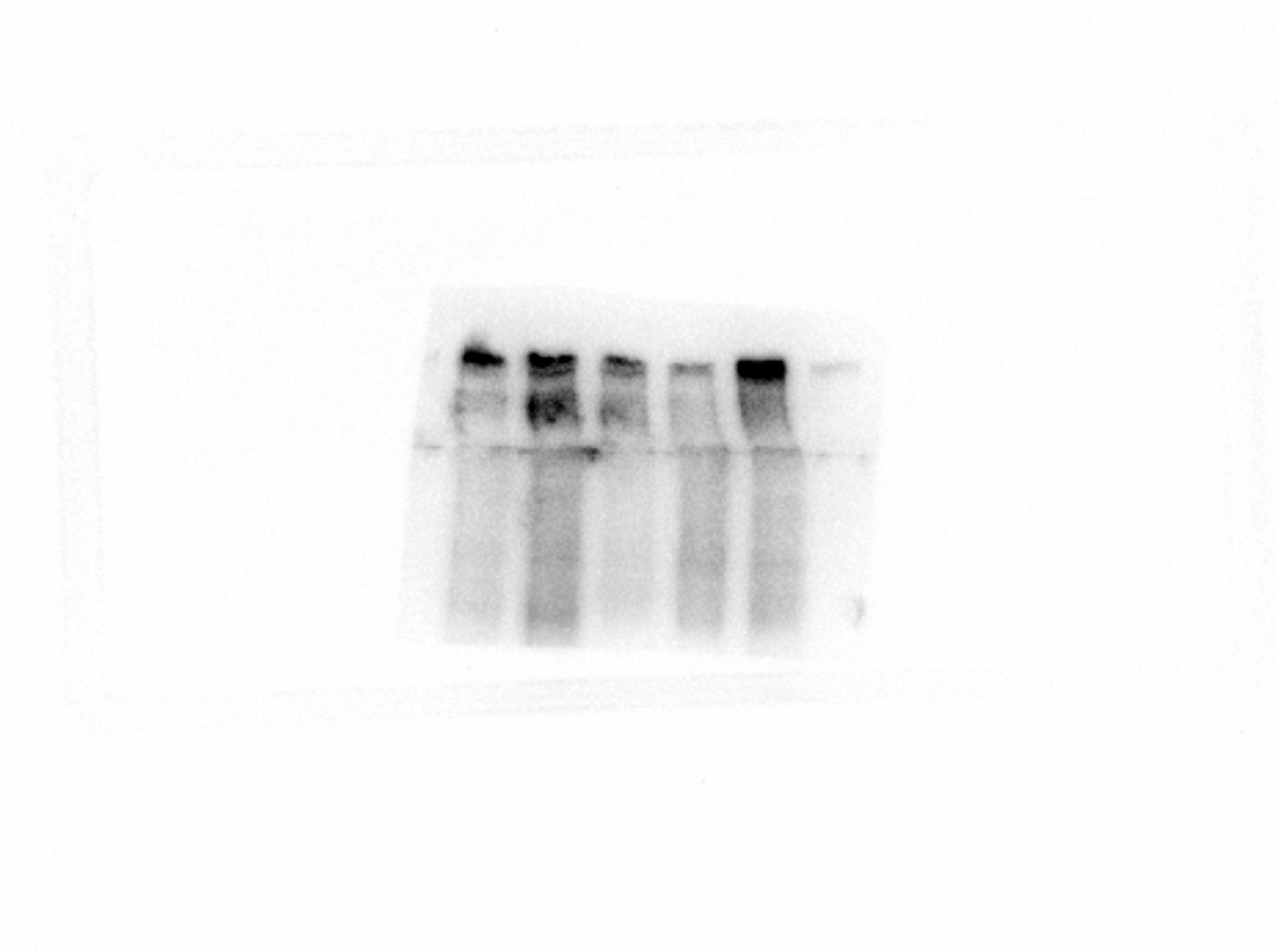

Supplement: Supplementary file 4 — Additional file 4. [file 12958_2022_988_MOESM4_ESM.zip › Fig. 7A--Axin2.tif]

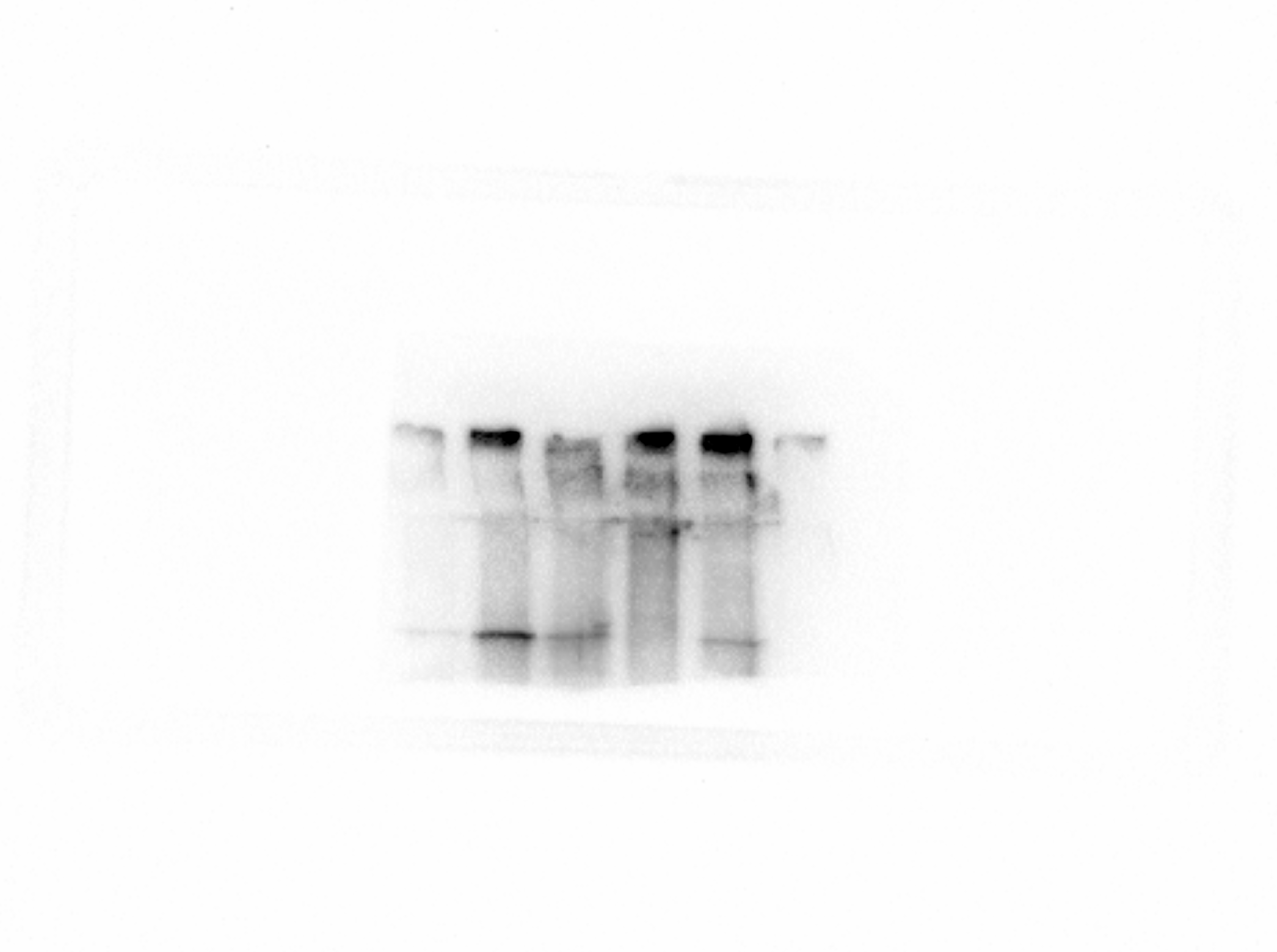

Supplement: Supplementary file 4 — Additional file 4. [file 12958_2022_988_MOESM4_ESM.zip › Fig. 7A--catenin.tif]

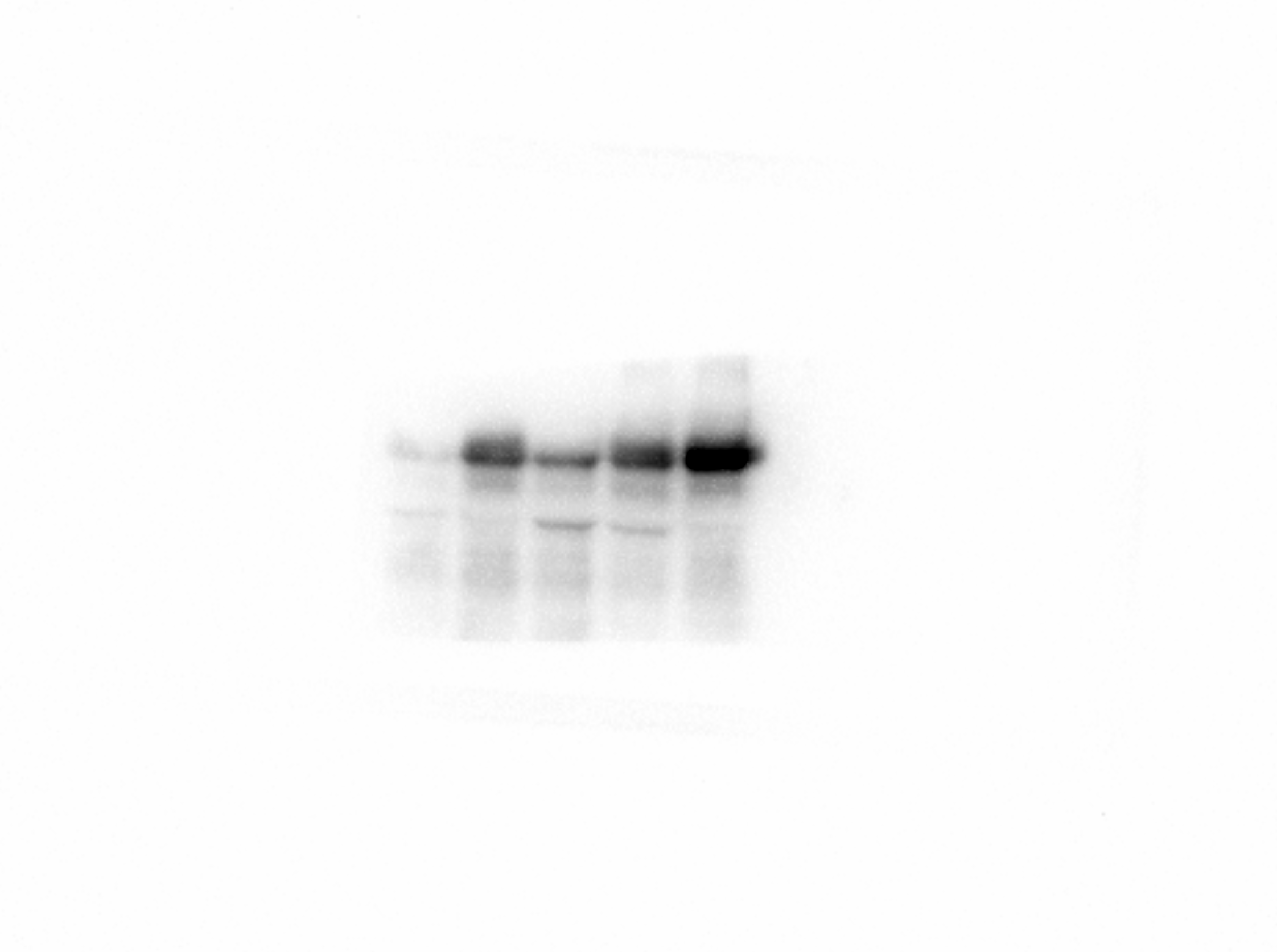

Supplement: Supplementary file 4 — Additional file 4. [file 12958_2022_988_MOESM4_ESM.zip › Fig. 7A--C-myc.tif]

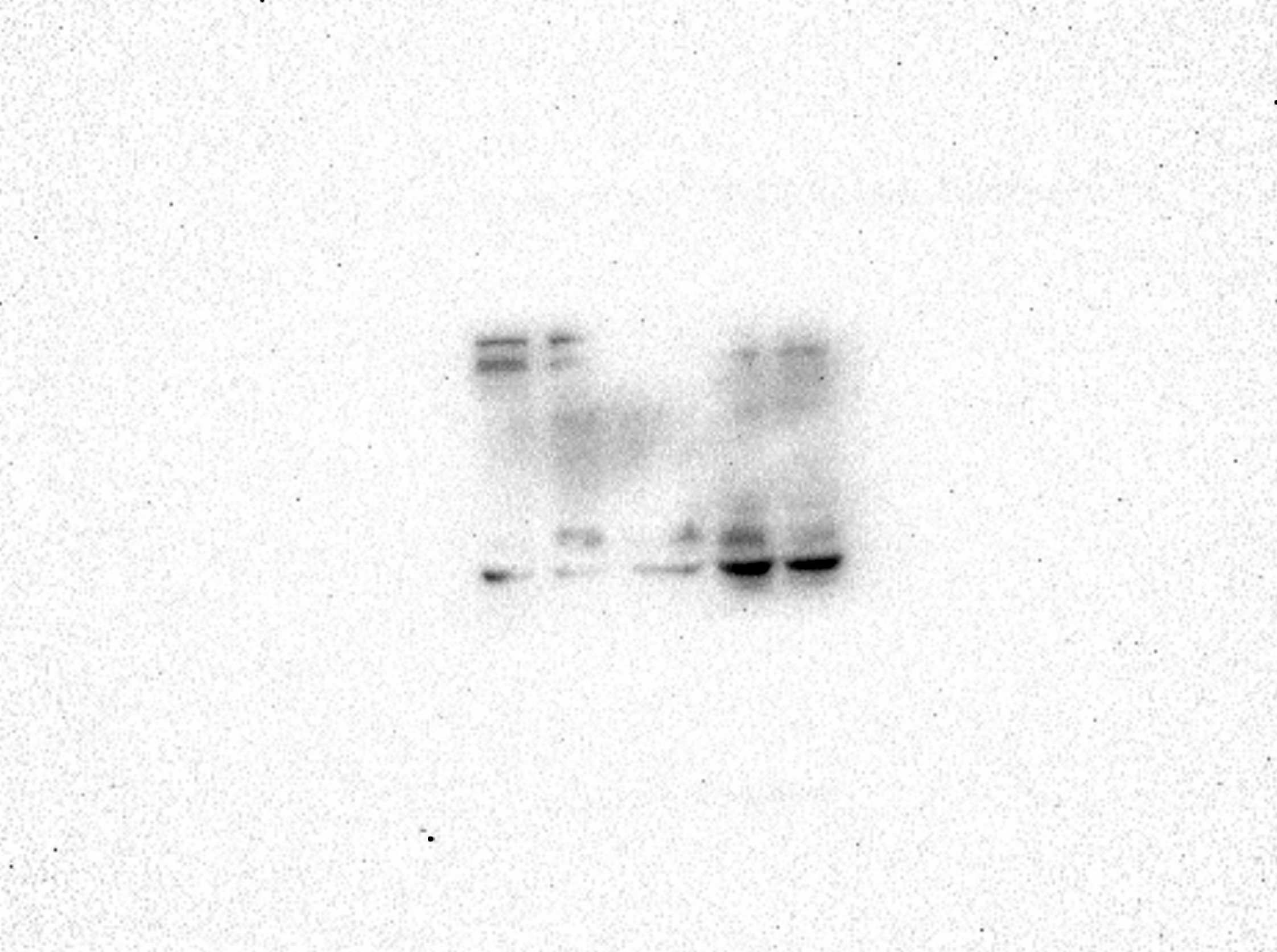

Supplement: Supplementary file 4 — Additional file 4. [file 12958_2022_988_MOESM4_ESM.zip › Fig. 7A--CyclinE.tif]

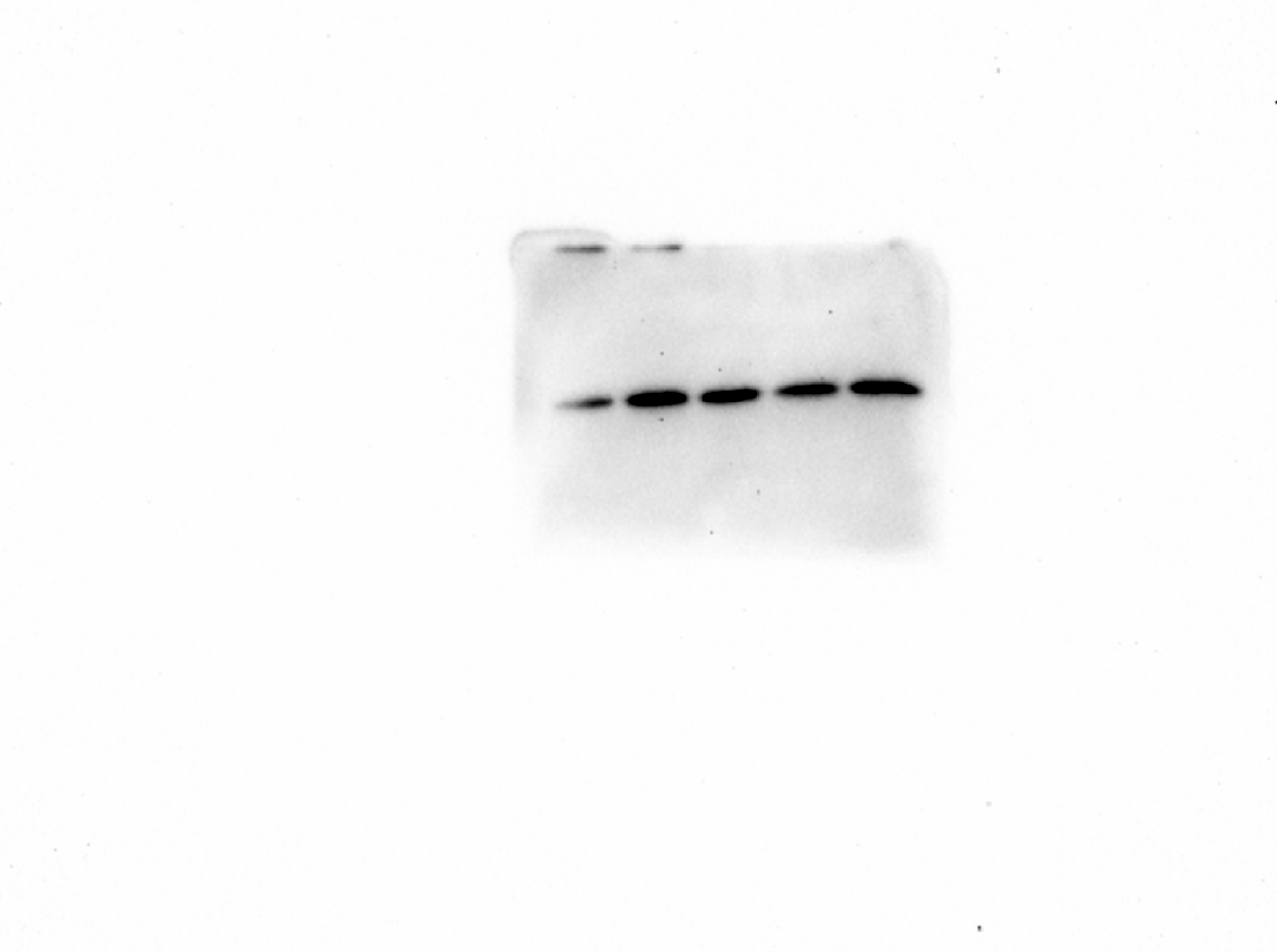

Supplement: Supplementary file 4 — Additional file 4. [file 12958_2022_988_MOESM4_ESM.zip › Fig. 7A--GAPDH.tif]
