# Supplementary material for: Bone marrow mesenchymal stem cells combined with estrogen synergistically promote endometrial regeneration and reverse EMT via Wnt/β-catenin signaling pathway
Source: Reprod Biol Endocrinol. 2022 Aug 15;20:121. doi: 10.1186/s12958-022-00988-1 (PMC9377128; doi:10.1186/s12958-022-00988-1)
Supplement: Supplementary file 5 — Additional file 5. [file 12958_2022_988_MOESM5_ESM.docx]

Dear Editors and Reviewers:

Thank you for giving us an opportunity to revise our manuscript. We appreciate the editors and reviewers very much for their constructive comments and suggestions on our original manuscript entitled “The synergistic effect of bone marrow mesenchymal stem cells combined with estrogen on repairing rabbit endometrial injury and inhibiting EMT via Wnt/β-catenin pathway”. Now we have revised the title of the manuscript to “Bone marrow mesenchymal stem cells combined with estrogen synergistically promote endometrial regeneration and reverse EMT via Wnt/β-catenin pathway”. These comments are very valuable and helpful for revising our paper. We have studied the comments carefully and made corrections on the original manuscript. All revised portions are marked in red in the revised manuscript. We hope you can consider the revision and look forward to hearing from you soon.

**Response to Reviewers**

**Reviewer #1:**

**Comments to the Author**

**This study aimed to investigate the therapeutic effects of BMSCs and estrogen in alleviating the endometrial damage-induced fibrosis, and explore the associated effects on endometrial fibrosis and EMT through Wnt/β-catenin signaling pathway. Intrauterine adhesions is an understudied and frustrating condition, and its pathophysiology is poorly understood. This study adds important knowledge by investigating the therapeutic effects of BMSCs and estrogen on endometrial healing providing mechanistic insights.**

**1. Abstract & Introduction: The authors state that “intrauterine adhesions (IUA) caused by endometrial injury is one of the main causes of female infertility”. This is a gross exaggeration. While the exact incidence of IUA is debatable, it is well known that IUA is a overall rare cause of infertility.**

Response: Thank you for the valuable suggestion. We have carefully modified and added the content about the description of IUA in the Abstract & Introduction part. The details of the revision have been highlighted in the revised manuscript.

**2. Methods, page 5: The authors state “the degree of cell fusion reached 80%”. They probably meant degree of cell confluence, not fusion.**

Response: Thank you very much for pointing out the problem. We have modified “the degree of cell fusion reached 80%” to “the degree of cell confluence reached 80%”.

**3.** **Why was LPS agent administered together with mechanical uterine injury? Was mechanical uterine injury alone not sufficient to induce a uterine phenotype?**

Response: Intrauterine adhesion is caused by endometrial basal layer injury owing to curettage or hysteroscopic surgery infection. Mechanical injury and infection are major factors inducing IUA, so the IUA rabbit model was generated by a dual damage method of mechanical curettage and lipopolysaccharide (LPS) infection. Mechanical curettage simulated clinical mechanical injury and LPS infection simulated clinical infection injury.

Through literature review, we have found that many studies used the method of double damage to construct the IUA animal models (PMID: 28723687; PMID: 31933162). Moreover, Fang Liu et al. found that compared with mechanical uterine injury, the double damage method was more stable for subsequent studies (PMID: 23199550).

4. **For the animal model, was endometrial injury performed on both uterine horns or just on one of the horns?**

Response: The endometrial injury performed on both uterine horns for the animal model. On the one hand, we selected the same number of rabbit uterus in the same batch as the control. On the other hand, during the surgical operation, if one side of the uterine horn was not ideal, the bilateral was more guaranteed for the experiment. At the same time, when collecting tissue samples, bilateral uterine horns can be used more for subsequent experimental studies.

**5. Figure 1D: In all the graphs, isotype controls are missing. These should be shown alongside the positive populations. Also, for CD45 and CD34 markers, it appears that the majority of the population falls within the marked area, yet the percentage states <2% which does not make sense. Did the authors mean to mark a different subset in the graph?**

Response: Thank you very much for pointing out the problem for us. We apologize for the incorrect flow cytometric image in Figure 1 (Fig. 1D). We have revised the picture as required and added isotype controls.

**6.** **The authors describe that the labeled BMSCs are present in the uterine tissues on days 3, 5 and 7 post-injury, but that the numbers of BMSCs are clearly decreasing over time both numerically and in location (initially periglandular and stromal and then only stromal on day 7). Did the authors evaluate whether these BMSCs persist at later time points that they have examined as well (e.g. 2 weeks and 3 weeks post-injury)? What is the fate of these cells? Do these cells functionally integrate in the tissue or only contribute transiently in a paracrine manner? This is an important point to make. A prior paper by Yi et al. (PMID: 30084961) demonstrated that transplanted BMSCs do not integrate into the tissue but rather provide a paracrine contribution. This point needs to be clarified and discussed in light of prior literature.**

Response: Thank you very much for your valuable comments. We labeled BMSCs with PKH26 fluorescent dye to track the number and distribution of transplanted BMSCs in vivo. During the experiment, we chose different time points (3d, 5d, 7d, 2 weeks, 3 weeks) to observe the localization of stem cells in vivo, and found that PKH26-labeled BMSCs mainly located in a small number of glands and surrounding matrix after orthotopic transplantation 3d and 5d. The transplanted BMSCS gradually decreased and mainly distributed in the endometrial stromal area in 7d. However, fluorescence intensity is very weak at 2 weeks, so there was no way to track the location and distribution of BMSC. Therefore, the relevant results are not reflected in the article.

This is an interesting question about whether BMSC is integrated into uterine tissue or only contribute transiently in a paracrine manner. Our results showed that estrogen plays an important role in supporting and improving utilization rates of BMSCs in the repair of endometrium, most likely by promoting the migration and differentiation of BMSCs into endometrial epithelial cells. At the same time, BMSCs decreased gradually with the extension of transplantation time. However, it is interesting to note that the effect of BMSCs on endometrial regeneration was more significant at 2 weeks and 3 weeks after orthotopic transplantation than at 1 week. Therefore, these results also indicate that BMSCs may promote endometrial repair through secreting other factors temporarily. In addition, the follow-up study found that the addition of estrogen after transplantation of BMSCs can significantly improve the ability of endometrial repair, which may be due to the fact that estrogen may provide a suitable environment for BMSCs to secrete related factors. The detailed description will be presented in the discussion of the revised manuscript.

**7. It is concluded that BMSCs combined with estrogen therapy further promoted endometrial repair by inhibiting the EMT occurrence. Vimentin is a stromal marker and its presence in the IUA group in the stromal area is expected. Its lack in the stroma of BMSC and/or estrogen treated groups does not specifically imply lack of EMT. To specifically refer to EMT (or lack thereof), one needs to demonstrate vimentin and/or mesenchymal marker staining in the epithelial compartment (i.e. luminal or glandular epithelium).**

Response: Thank you for your kind suggestion. Our results indicate that BMSCs combined with estrogen therapy further promoted endometrial repair by inhibiting the EMT occurrence. We detected the relative expression of epithelial marker (E-cadherin, CK7) and mesenchymal marker (N-cadherin, Vimentin and ZEB1) by Western blot and immunofluorescence staining.

After constructing the IUA model, the uterine morphology was destroyed, accompanied by decrease of glandular and luminal epithelium and increase of stroma, so the positive rate of CK7 staining was reduced, while the vimentin was increased. However, the BMSC and estrogen treatment group restored endometrial morphology and increased the number of glands, so the opposite results were shown.

We detected the expression of CK7 and vimentin in normal endometrial epithelium and stroma respectively, and found that CK7 was only positive in the epithelium, and vimentin was only positive in the stroma.

**8. There are multiple English grammatical and syntax errors throughout the manuscript. The manuscript must undergo editing by a native English speaker.**

Response: We apologize for the poor English grammatical and syntax errors in our manuscript. The revised version was edited and made the appropriate modifications by a native English speaker.

Once again, thank you very much for your constructive comments and suggestions. We tried our best to make some changes to improve our manuscript. We appreciate for Editors and Reviewers and hope that the correction version will meet with approval. We deeply appreciate your consideration of our manuscript.

Best wishes.

Yours sincerely,

Dan Liu
E-mail: nxld@163.com
